# Advancements in Wearable and Implantable BioMEMS Devices: Transforming Healthcare Through Technology

**DOI:** 10.3390/mi16050522

**Published:** 2025-04-28

**Authors:** Vishnuram Abhinav, Prithvi Basu, Shikha Supriya Verma, Jyoti Verma, Atanu Das, Savita Kumari, Prateek Ranjan Yadav, Vibhor Kumar

**Affiliations:** 1 Department of Electrical Engineering, Indian Institute of Technology Bombay, Mumbai 400076, Maharashtra, India; vishnuram.abhinav@gmail.com; 2 Department of Electrical Engineering, Texas A&M University, College Station, TX 77843, USA; 3 Integrated Disease Surveillance Program, National Health Mission, Guwahati 781005, Assam, India; 4 Department of Electronics and Communication Engineering, Manipal Institute of Technology, Manipal Academy of Higher Education, Manipal 576104, Karnataka, India; 5 Department of Biosciences and Bioengineering, Indian Institute of Technology Bombay, Mumbai 400076, Maharashtra, India; 6 School of Mechanical and Materials Engineering, University College Dublin, D04 V1W8 Dublin, Ireland

**Keywords:** bioMEMS, wearable medical devices, implantable devices, healthcare monitoring, chronic disease management, point of care, drug delivery, cardiac monitoring, societal determinants, biocompatible materials

## Abstract

Wearable and implantable BioMEMSs (biomedical microelectromechanical systems) have transformed modern healthcare by enabling continuous, personalized, and minimally invasive monitoring, diagnostics, and therapy. Wearable BioMEMSs have advanced rapidly, encompassing a diverse range of biosensors, bioelectronic systems, drug delivery platforms, and motion tracking technologies. These devices enable non-invasive, real-time monitoring of biochemical, electrophysiological, and biomechanical signals, offering personalized and proactive healthcare solutions. In parallel, implantable BioMEMS have significantly enhanced long-term diagnostics, targeted drug delivery, and neurostimulation. From continuous glucose and intraocular pressure monitoring to programmable drug delivery and bioelectric implants for neuromodulation, these devices are improving precision treatment by continuous monitoring and localized therapy. This review explores the materials and technologies driving advancements in wearable and implantable BioMEMSs, focusing on their impact on chronic disease management, cardiology, respiratory care, and glaucoma treatment. We also highlight their integration with artificial intelligence (AI) and the Internet of Things (IoT), paving the way for smarter, data-driven healthcare solutions. Despite their potential, BioMEMSs face challenges such as regulatory complexities, global standardization, and societal determinants. Looking ahead, we explore emerging directions like multifunctional systems, biodegradable power sources, and next-generation point-of-care diagnostics. Collectively, these advancements position BioMEMS as pivotal enablers of future patient-centric healthcare systems.

## 1. Introduction

The BioMEMS industry, projected to reach USD 24.5 billion by 2030, has revolutionized healthcare by integrating miniaturized biomedical devices, real-time physiological monitoring, and digital health platforms. Wearable and implantable BioMEMSs now form the technological backbone of this revolution, enabling continuous biomarker tracking, closed-loop diagnostic systems, and adaptive therapeutic delivery. BioMEMS devices enable personalized diagnostics, targeted therapy, minimally invasive interventions, and real-time monitoring—driving better patient outcomes and streamlined healthcare delivery [1].

BioMEMSs are microscale systems that integrate biosensors, actuators, microfluidics, wireless communication, and signal processing within a compact and biocompatible platform. They leverage innovations in materials science, microfabrication, and electronics to interact with biological systems in minimally invasive ways. BioMEMSs can be divided into two major domains that depend on their application and form factor. The first is wearable and the second is implantable devices. A wearable BioMEMS provides non-invasive and external monitoring of physiological parameters and biochemical markers [2]. An implantable BioMEMS works within the body of the subject, which enables continuous internal sensing, targeted drug delivery, and neurostimulation [3]. In combination, both technologies play an important role in moving the modern healthcare system to a more proactive, real-time, and personalized approach that emphasizes inhibition and early intervention over reactive treatment.

Due to their versatility, accessibility, and ease of integration into daily life, wearable BioMEMSs have attracted lots of attention. Wearable devices—for example, biosensor-integrated wristbands, patches, microneedle arrays, smart textiles, etc.—are designed to monitor parameters like heart rate, respiration, glucose levels, hydration, muscle activity, and movement [4]. In these devices, the sensing of electrochemical, optical, and mechanical parameters is the backbone. Sensing of those parameters provides accurate and consistent monitoring of the physiological signals [5,6]. The combination of wearable sensors with flexible substrates and wireless units has enabled the development of highly conformal and user-friendly systems. Those systems are very useful in transmitting the data of the body to smartphones or cloud platforms for real-time health monitoring [7].

Conversely, an implantable BioMEMS provides highly accurate and focused functionalities, specifically for patients that need regular monitoring or intervention. Implantable devices include glucose monitoring systems, pressure sensors for cardiac monitoring, neural stimulators, and programmable drug delivery devices [8,9]. As implantable devices work directly inside the body, they can overcome the limitations of external monitoring. Therefore, they are great candidates to provide precise, independent, and long-lasting solutions for overseeing complex health issues, and offer a more effective way to care for patients over time. Developments in designing parts like power-efficient designs, encapsulation within biocompatible materials, and wireless telemetry have further improved the performance and lifetime of implantable devices [10,11]. Due to these developments, implantable BioMEMSs are increasingly implemented in diabetes management, epilepsy control, cardiovascular monitoring, cancer treatment, and neurorehabilitation.

The ongoing innovations in the BioMEMS area is based on progress in some foundational areas. Among them are the development of novel materials like conductive polymers, stretchable nanocomposites, biodegradable substrates, and hydrogels, which have enhanced the flexibility of devices, their durability, and their integration with soft tissues [12]. These novel materials also provide benefits in minimizing immune responses, enhancing the quality of the signal, and allowing for short-term implants that dissolve after completing their purposes. Additionally, advances in sensor design have allowed for multimodal detection of physical, chemical, and electrophysiological signals, often within a single device. The miniaturization of these sensors, combined with energy-efficient wireless communication and signal processing algorithms, has made continuous monitoring feasible without compromising user comfort or safety [8].

Another important dimension of BioMEMS innovation lies in the expanding clinical applications. In chronic disease management, wearable and implantable systems are being used to continuously monitor cardiovascular metrics, respiratory parameters, glucose levels, and neurological activity, providing both patients and clinicians with actionable insights [13]. In cardiology, wearable ECG monitors and implantable pressure sensors facilitate early detection of arrhythmias [14], hypertension, and heart failure episodes [15]. Respiratory health monitoring has benefited from wearable respiratory rate sensors and lung sound detectors that enable the early identification of exacerbations in asthma and chronic obstructive pulmonary disease (COPD) [16]. Similarly, intraocular pressure sensors, both wearable and implantable, are revolutionizing glaucoma management by offering continuous monitoring and reducing the need for frequent clinical visits [17].

In addition to diagnostics, therapeutic applications of BioMEMSs are gaining momentum. Implantable drug delivery systems allow precise, localized, and programmable administration of medications, reducing systemic side effects and improving patient compliance [18]. Such systems have played a significant role in pain management, cancer therapy, and hormone regulation. Neurostimulation devices like deep brain stimulators, vagus nerve stimulators, and spinal cord stimulators utilize electrical pulses to modulate neural activity. As a result, they offer treatment alternatives for neurological disorders, chronic pain, and mental health conditions [19,20]. The integration of sensing and stimulation with a BioMEMS provides a method of intelligent, self-regulating medical interventions.

The coupling of the BioMEMS area with AI, machine learning (ML), and the IoT further enhances its influence [21]. AI algorithms can analyze a wide variety of physiological data acquired by BioMEMS devices, which is useful in detecting various patterns, predicting adverse events, and personalizing treatment regimens [22]. IoT connectivity provides remote monitoring of the patient, real-time alerts, and smooth communication between patients, caregivers, and healthcare companies. Such implementation of digital technologies in BioMEMSs provides intelligent health systems that are capable of delivering precise, proactive care in both clinical and home environments [23].

Despite these great developments, there are still some challenges that need to be addressed for the adoption of BioMEMSs. The most significant challenge, among others, is the energy supply [24]. Most BioMEMS devices rely on batteries, which are bulky, have a limited lifespan, or require frequent recharging. In this quest, significant research is being carried out in the area of energy-harvesting technologies, like piezoelectric nanogenerators, triboelectric sensors, and biofuel cells. Those systems can harness energy from physical motion, thermal gradients, or biochemical reactions. Another big challenge is maintaining data accuracy and reliability over a long time period. Biological noise, sensor drift, and environmental impacts can degrade the quality of the signal, which necessitates the development of advanced calibration algorithms, robust sensor design, and ML-based error correction techniques [25].

Biocompatibility and long-term stability are highly important for implantable devices. When these systems are exposed to physiological fluids, they can experience corrosion, biofouling, and inflammatory reactions. As a result, the device performance may become degraded/obsolete or necessitate surgical removal [26]. In order to address such issues, the scientific community is working on the development of advanced coatings like antifouling polymers, zwitterionic surfaces, and bioresorbable materials, which are helpful in reducing immune responses and enhancing the device’s durability [27]. Moreover, temporary or short-term implants that are made up of degradable materials are now becoming attractive for implementing in post-surgical monitoring and controlled drug delivery. This is because they eliminate the need for retrieval procedures [28].

In addition to technical challenges, regulatory and societal factors have a significant influence on the development of BioMEMSs [29]. The approval process for medical devices requires rigorous safety and efficacy evaluations, which vary by region or country. This absence of unified global standards adds further obstacles to achieving commercial scalability and clinical acceptance. Furthermore, issues of data privacy, cybersecurity, and patient acceptance are becoming increasingly important as BioMEMSs become part of digital health ecosystems. Ensuring secure data transmission, regulatory compliance with HIPAA or the GDPR, and culturally sensitive design will be crucial for building public trust and fostering widespread adoption [30].

This review presents a comprehensive and structured analysis of recent advancements in wearable and implantable BioMEMS technologies, emphasizing their growing role in transforming healthcare delivery. It examines innovations in biocompatible and biodegradable materials, advanced sensing modalities, low-power operation, wireless data transmission, and device miniaturization—key enablers of next-generation BioMEMS systems. The review categorizes BioMEMS applications into diagnostic, therapeutic, and monitoring domains, covering use cases such as chronic disease management, neurostimulation, targeted drug delivery, and continuous physiological monitoring.

Additionally, it addresses the integration of BioMEMSs with digital health ecosystems, including AI and IoT frameworks, which are enhancing data-driven decision-making and enabling personalized medicine. Alongside these advancements, the review critically evaluates existing challenges, such as regulatory complexity, global standardization gaps, power limitations, long-term biocompatibility, and societal acceptance. By synthesizing both current developments and future directions, this review highlights the transformative potential of BioMEMS technologies in enabling smarter, more responsive, and patient-centric healthcare systems, while also outlining the roadmap for their successful clinical translation and large-scale adoption.

## 2. Materials for BioMEMS Devices

BioMEMS devices require materials that are not only miniaturizable and mechanically flexible but also biocompatible, stable in physiological environments, and suitable for integration with electronic and microfluidic components. The choice of material affects the performance, durability, signal quality, and patient comfort. Broadly, materials used in bioMEMSs can be classified into synthetic polymers, biodegradable and bioresorbable materials, natural polymers, and emerging hybrid materials. Each class provides different advantages based on the desired application—for long-term implantation, transient diagnosis, or disposable wearables. This section discusses representative materials from these categories, focusing on their structural, chemical, and biological properties that make them suitable for bioMEMS application.

### 2.1. Synthetic Polymers

#### 2.1.1. Polydimethylsiloxane (PDMS)

Polydimethylsiloxane (PDMS) is a silicone-based polymer with unique physicochemical properties. It is frequently used in MEMS, microfluidics, and bioMEMS applications. The structure of PDMS consists of repeating (CH_3_)_2_SiO units, wherein the Si-O backbone provides flexibility and adjustable mechanical properties. It can be synthesized with varying levels of cross-linking that allows PDMS to exist in various forms, including fluids, emulsions, lubricants, resins, elastomers, and rubbers [31]. The surface methyl groups in PDMS lead to its low intermolecular interactions, which contributes to its lower surface tension and hydrophobic nature, and therefore make it well suited for advanced applications [32].

One of the best advantages of PDMS is its outstanding chemical stability. It does not react with many acids, bases, or solvents, which makes it suitable for applications where chemical resistance is important. Moreover, PDMS can withstand temperatures ranging from −40 °C to 250 °C without notable degradation [33,34,35]. PDMS is also well known for its optical transparency across a broad wavelength range (240–1100 nm), making it a most suitable material for many optical and bioimaging applications. Its transparency along with its refractive index (~1.41) leads to minimal light scattering. This property is beneficial specifically for microfluidic devices requiring optical detection [36]. Another crucial property is its biocompatibility, which allows PDMS to be widely used in biomedical devices, BioMEMS, and implantable sensors. It exhibits minimal cytotoxicity [37] and supports cell adhesion when surface-modified. Such a property makes it suitable for direct interaction with biological tissues. Additionally, the gas-permeability of PDMS is great, which allows for efficient oxygen exchange. Therefore, it is beneficial in cell culture applications and bioengineering [38,39,40].

Mechanically, PDMS is extremely stretchable and flexible. Depending on the cross-linking ratio, it has an elongation at break exceeding 100% [41]. The Young’s modulus for this material can be changed from a few kPa to several MPa [41], which enhances its applicability in a diverse range of applications, i.e., from soft electronics to structural support in flexible devices. The low elastic modulus allows PDMS to undergo significant deformations without losing its structural integrity, making it suitable for many wearable electronics [42]. The unique adhesion property of PDMS allows it to selectively bond to various substrates through oxygen plasma treatment, thermal curing, or chemical modification. It demonstrates separate adhesion and non-adhesion areas under UV exposure, which are very helpful in microfabrication and patterning [33,34].

Additionally, PDMS is a perfect fit for composite materials. It has the ability to integrate with many conductive fillers, such as metal nanoparticles, carbon-based nanomaterials, and conductive polymers, enhancing its electrical properties while keeping its mechanical flexibility. It has been successfully combined with liquid metal alloys such as eutectic gallium–indium (EGaIn), which significantly improves its electrical permittivity while maintaining elasticity. Additionally, PDMS has been combined with thin metal films composed of titanium and gold, as well as silver-based nanostructures, including silver nanowires and nanoparticles, further expanding its functional capabilities [42,43,44,45,46]. One of PDMS’s notable characteristics is its hydrophobicity, which makes it particularly suitable for applications exposed to moisture. Researchers have developed methods to enhance this property, creating superhydrophobic PDMS surfaces with contact angles exceeding 150° and low hysteresis [47].

#### 2.1.2. Polyimide

Polyimides (PIs) are another well-known class of synthetic polymers. PIs can tolerate very high temperatures without losing their strength or form because they are very resilient. They are also ideal for protecting fragile devices because of their superior electrical insulating qualities. PIs are the preferred option for performance and dependability in high-tech applications and harsh environments. Polyimides have aromatic heterocyclic chains with alternating carbonyl (C=O) and nitrogen (N) groups, and they form a charge transfer complex. Such a structure makes polyimides chemically inert, structurally rigid, and resistant to chemical degradation. Due to the above properties, PIs are very durable in challenging biological environments [32,48]. Their synthesis involves a two-step process where a dianhydride and a diamine react in a dipolar aprotic solvent like N,N-dimethylacetamide (DMAc) or N-methylpyrrolidone (NMP), resulting in polyamic acid that undergoes cyclization to form the final PI [49].

Exceptional mechanical properties of PIs, like a tensile strength of 78.3 ± 7.3 MPa [50], an elongation at break of 7.4% [41], and a Young’s modulus of 2.5 GPa [51], make them great candidate for use in bioMEMS devices that require flexibility, durability, and strength. The low density (1.33 g/cm^3^) of PIs [52] further enhances their applicability in medical applications, as they are lightweight while still providing excellent mechanical strength. These materials show a glass transition temperature (T_g_) of 325 °C [53], which is notably higher than that of many other polymers. This property allows them to maintain their molecular structure in a high-temperature environment [54]. Beyond their thermal stability, polyimides are biocompatible, meaning they can safely interact with biological tissues without causing adverse reactions. They also exhibit excellent chemical resistance and a high degree of cytocompatibility, making them ideal for use within the human body [48,49]. Their superior dielectric properties, with a dielectric constant of 3.4, make polyimides an excellent choice for electrical insulation in bioelectronic devices such as biosensors and neural electrodes [54]. Additionally, they resist oxidation and absorb very little water, ensuring stability in physiological environments. These combined properties make PIs particularly well suited for applications in implantable medical devices, where durability and compatibility with biological systems are paramount.

#### 2.1.3. Parylene C

Parylene C is a highly versatile material with a unique combination of mechanical, thermal, electrical, and biocompatible properties, making it ideal for applications such as biomedical implants, microelectronics, and wearable devices. It boasts a tensile strength of ~70 MPa [31,55], which provides significant resistance to mechanical stress. Furthermore, its elongation at a break of around 200% [56] demonstrates its moderate flexibility and toughness. This flexibility allows parylene C to conform seamlessly to complex surfaces without cracking or compromising its structural integrity.

These characteristics make parylene C particularly well suited for protective coatings in challenging environments. Its ability to maintain durability and adaptability ensures reliable performance in applications requiring both strength and precision. Its Young’s modulus of 4.5 GPa [56] and Shore D hardness ranging from 35 to 80 [55] suggest a balance between its rigidity and elasticity. Due to this, it is ideal for coatings on flexible substrates, particularly in microfabrication and biomedical applications [57,58].

Parylene C exhibits a low density of 1.289 g/cm^3^, allowing for minimal weight addition when applied as a thin film coating. Its hydrophobic nature is demonstrated by its remarkably low water absorption rate of 0.1% after 24 h of immersion, making it ideal for use in humid environments and medical implants [55]. The material’s durability is noteworthy, as it resists swelling and degradation when exposed to bodily fluids and harsh chemicals [59,60]. While its biodegradability is moderate, parylene C demonstrates high cytocompatibility and hemocompatibility, rendering it suitable for long-term biomedical applications such as cardiovascular stents, neural probes, and peripheral nerve electrodes [57,60,61].

Parylene C also exhibits excellent resistance to oxidation and UV radiation, ensuring its stability in outdoor applications and as a protective coating [60,62]. Its thermal properties are also noteworthy, with a glass transition temperature of 35–80 °C, a melting point of approximately 290 °C [55], and a relatively low thermal conductivity of 1.0 W/m·K [55], making it suitable for use in thermally sensitive applications [32,63].

Electrically, parylene C functions as an excellent insulator, boasting a dielectric constant of 2.95 at 1 MHz [55]. This characteristic, combined with its moisture resistance, makes it highly suitable for use in coatings and implantable bioelectronic devices where electrical insulation and environmental protection are critical [57,62]. Furthermore, parylene C demonstrates high optical transparency in certain wavelengths, enabling its application in various optical and biosensing devices that require minimal light attenuation [58,60]. The ability to deposit ultra-thin, defect-free layers with high adhesion to various substrates has expanded its use in implantable medical devices and other advanced applications [32,59,63].

#### 2.1.4. Poly(methyl methacrylate) (PMMA)

Poly(methyl methacrylate) (PMMA) is a durable and versatile polymer with mechanical properties that make it particularly valuable in biomedical and wearable technologies. Its tensile strength of 60–70 MPa [64] ensures reliable performance under mechanical stress, enabling long-term durability in medical devices such as prosthetics and dental implants [65]. The material’s limited stretching capacity (0.5–5% elongation at break) [64] prevents excessive deformation under strain, preserving structural integrity in applications requiring dimensional stability [65]. With a Young’s modulus of 3.3 GPa [66], PMMA exhibits stiffness comparable to some metals, making it suitable for applications where rigidity and resistance to bending are critical. This rigidity is complemented by a high surface hardness (Shore D 96), which minimizes wear and scratches in high-contact environments like orthopedic devices or optical components [65].

At a density of 1.17 g/cm^3^, PMMA is lightweight, a key advantage for wearable devices where user comfort and ergonomic design are priorities [67]. While it does not absorb water significantly, PMMA’s biodegradability is minimal, which is advantageous for its use in long-lasting implantable devices [65]. Its cytocompatibility further enhances its suitability in medical applications, ensuring that it does not cause adverse biological responses [68]. PMMA also shows strong oxidation resistance and is capable of withstanding UV exposure, making it ideal for environmentally stable applications [65].

With a glass transition temperature of 105 °C [69], PMMA maintains its mechanical properties over a wide range of temperatures, making it reliable in both ambient- and elevated-temperature environments. Its relatively high melting point of ~166 °C [70] ensures that it remains stable during manufacturing processes and in use. The thermal conductivity of PMMA is 0.22 W/mK [67], indicating that it does not dissipate heat quickly, a property that can be beneficial in thermally sensitive applications.

The dielectric constant at 1 MHz is 3.5 [71], suggesting its insulating properties and making it suitable for electrical applications in devices. Moreover, its electrical conductivity is extremely low (1 × 10^−19^ S/m) [72], making it a good insulator, which is beneficial in applications requiring minimal electrical interference. PMMA possesses a remarkable optical transparency that significantly enhances its utility in medical and diagnostic applications where visual clarity is paramount [65]. This property allows for the creation of devices that can be easily monitored and inspected, facilitating more accurate diagnoses and treatments.

#### 2.1.5. Polyurethane (PU)

Polyurethane (PU) has emerged as a remarkable material in modern medicine, combining nature-inspired design with cutting-edge engineering. Its structure, which mimics the peptide bonds found in proteins, allows it to seamlessly integrate with the human body [12]. The structure of PU consists of hard segments made from diisocyanates and small molecule chain extenders like diols or diamines, and soft segments made of oligomeric polyols [73]. This unique composition, featuring both rigid and flexible segments, enables PU to adapt to various medical needs while exhibiting properties like anti-aging, antibacterial, and self-healing capabilities.

PU boasts a high tensile strength of 18 MPa [74], as a result of which it can withstand significant forces without breaking. Its ability to stretch to more than 140% of its original length before breaking demonstrates its exceptional elasticity [75]. Furthermore, its Young’s modulus, ranging from 3.6 to 88.8 GPa, indicates a versatile stiffness that can be tailored for various medical applications [75]. For instance, researchers have developed electrospun PU wound dressings that incorporate a color-changing probe to detect bacterial infections early. This smart dressing leverages PU’s flexibility to conform to the body’s contours while maintaining its structural integrity, even as it performs its vital monitoring function [76].

Most excitingly, PU’s self-healing capabilities are opening new frontiers in wearable technology and health monitoring. The self-healing PU elastomer, which operates through dynamic aromatic disulfide exchange and strong hydrogen bonding, can recover its electrical conductivity even after being stretched by 60%. These capabilities ensure minimal performance loss and enhanced durability, which is vital for long-term usage [77]. PU’s thermal stability, with a glass transition temperature of −35 °C and a low thermal conductivity (0.19 ± 0.03 W/m·K) [78], also ensures its functionality in a broad range of environmental conditions without degrading. PU is biodegradable and biocompatible, ensuring its safe and efficient degradation within the body. The material’s high strength and flexibility, demonstrated by its ability to stretch up to 630% without breaking, are also crucial for its potential use in medical implants, such as heart valves, where durability and flexibility are paramount.

#### 2.1.6. Polypyrrole (PPy)

Polypyrrole (PPy) is an intrinsically conductive polymer that exhibits outstanding properties. Due to those properties, it is highly suitable for biomedical applications, particularly in wearable and implantable devices. Its high conductivity, environmental stability, and biocompatibility make PPy ideal for applications in bioelectronics and biomedicine [79]. It has reversible redox properties, which are beneficial for applications requiring rapid charge transfer, such as in bioelectronics [79]. Its ability to conduct electricity under physiological circumstances and its biocompatibility allow it to be used in nerve electrode interfaces and conductive coatings for tissue engineering [80,81].

Its electroactive behavior comes from the delocalization of electrons in its conjugated backbone, which facilitates conductivity when doped with halogenic electron acceptors [82,83]. However, PPy is also brittle and nondegradable, which can limit its use in some applications, such as long-term tissue engineering. Such issues can be resolved by incorporating PPy into composite materials with natural or synthetic biomaterials. Such processes can tailor PPy’s mechanical and electrical properties to suit specific biomedical requirements [80].

PPy’s synthesis can be achieved through electrochemical or chemical polymerization, with the latter method offering the advantage of bulk production and covalent modification [80]. Its conductivity can be significantly influenced by its morphology, which can be modified by the addition of surfactants such as sodium dodecyl sulfate (SDS) during polymerization, leading to enhanced conductivity through sheet-like structures [84]. The selection of the oxidizing agent plays a crucial role in determining PPy’s final conductivity. For instance, using ferric chloride (FeCl_3_) as an oxidant often results in higher conductivity compared to ammonium persulfate. This flexibility in synthesis allows researchers to tailor PPy’s electrical properties to suit specific needs [85]. One of PPy’s standout features is its impressive thermal stability. It can maintain its properties up to temperatures of 300 °C, making it a reliable option for applications that operate in high-temperature environments [82].

In the realm of biomedical applications, PPy exhibits intriguing antibacterial properties. These can be further enhanced through modification techniques such as oxygen plasma immersion ion implantation (O-PIII). This process improves surface wettability and charge, introducing functional groups like C-O/C=O bonds and protonated nitrogen. These modifications enhance PPy’s surface chemistry, thereby boosting its antibacterial efficacy [81]. The versatility of PPy extends to its ability to accommodate various dopants. By incorporating these additives, researchers can fine-tune PPy’s electrochemical and optical properties, expanding its potential applications in energy storage devices and sensors. However, like any material, PPy is not without its challenges. In its conventional bulk form, it struggles with poor solubility and limited mechanical ductility. Ingeniously, these issues can be mitigated by utilizing nanostructured forms of PPy. These nanostructures offer enhanced electrochemical and optical properties due to their larger surface area and well-defined nanotopography [86].

The combination of electrical conductivity, flexibility, and biocompatibility makes PPy an excellent candidate for wearable and implantable devices. Its ability to undergo reversible doping cycles also renders it suitable for various biosensors that require rapid charge and discharge capabilities. Furthermore, PPy’s morphological versatility, allowing it to form thin layers, nanoparticles, and nanofibers, further expands its potential applications [83]. The comparison of the properties of different synthetic polymers is shown in Table 1.

### 2.2. Biodegradable Polymers

#### 2.2.1. Polylactic Acid (PLA)

Polylactic acid (PLA) has emerged as a remarkable material in the field of medicine, offering a unique combination of sustainability and biocompatibility. This versatile thermoplastic polyester, with its roots tracing back to the 18th century, has evolved from a scarce and expensive substance to a widely used material in modern medical applications. PLA’s journey began with its humble origins as lactic acid, first isolated by the chemist Scheele in the 1700s. Over time, it has transformed into a biobased and biodegradable aliphatic polyester, aligning perfectly with today’s focus on environmentally friendly materials [109]. Initially, PLA’s use was limited to specialized medical applications due to its high cost and limited availability. However, advancements in production techniques have dramatically expanded its potential, allowing it to be processed into various forms through methods such as extrusion, injection molding, and electrospinning [110].

In recent years, the field of biomaterials has witnessed significant progress, particularly in the development of biodegradable and bioresorbable polymers. This progress, coupled with innovative bio-fabrication [111] methods, has led to a surge in the use of biopolymers like PLA. These materials are now finding applications not just in plastic bags but also in medical devices. As industries aim for more sustainable production practices to meet the growing demand for environmentally friendly polymers, PLA has gained recognition as a viable alternative [112,113]. PLA shares similar characteristics with polymers like polystyrene, polypropylene, and polyethylene terephthalate, broadening its potential use across various industries beyond medical applications.

PLA is a member of the poly-α-hydroxy acid family, which is a type of linear aliphatic thermoplastic polyester. The structure of PLA consists of repeating units of lactic acid monomers, which are derived from the fermentation of sugars. The basic monomer of PLA is lactic acid (C_3_H_6_O_3_), which has a hydroxyl group (-OH) and a carboxyl group (-COOH) [114]. The polymerization of lactic acid (LA) creates a long chain with ester linkages. PLA is synthesized through a condensation polymerization process (usually involving lactide intermediates), where water molecules are eliminated as the monomers bond, forming ester linkages.

LA is an important compound in the glycolytic cycle, discovered in 1881 through fermented milk extraction [115]. It exists as two optical isomers, D-LA (PDLA) and L-LA (PLLA). Commercial LA is produced by fermenting sugars like glucose and starch using bacteria, with traditional methods involving the addition of calcium compounds to form crude lactic acid. Purification processes such as dyeing, electrolysis, and crystallization yield lactic acid for medical and food applications [116].

For large-scale production, lactonitrile intermediates are used. PLA, made from LA, exists in optically active forms (D- and L-forms) and a meso-form, which is optically inactive. The synthesis of PLA involves multiple stages, with lactide formation as an intermediary step [117,118]. Various polymerization methods, like azeotropic dehydration condensation (ADC) and ring opening polymerization (ROP), are used to convert LA into PLA. PLA is a versatile material that is hydrophobic and offers desirable rigidity, making it ideal for biomedical devices. Key properties like degradation rate, cytotoxicity, tensile strength, and recrystallization rate are crucial for medical applications [119,120]. PLA can be molded into different structures, such as nanoparticles, films, scaffolds, and sutures, and is used in consumer goods, packaging, and medical devices. With rising demand for PLA in the healthcare sector, further research is needed to develop new technologies to enhance its use, particularly in prosthetics and cardiovascular devices [119].

The metabolism of PLA within the human body results in the intermediate product, lactic acid, which is non-toxic and non-hazardous, thus allowing it to be safely processed by the body [121]. This characteristic makes PLA ideal for a diverse array of medical uses, such as bone stents, wound dressings, masks, surgical gowns, absorbable vascular stents, heart valves, medical sutures, pharmaceutical slow-release systems, and other hygiene products for physiological applications [122]. PLA is frequently combined with other polymers, such as polyethylene glycol (PEG), polyglycolic acid (PGA), poly-l-glycolic acid (PLGA), poly(3-hydroxybutyrate-co-3-hydroxyvalerate) (PHBV), and PDLA, to enhance its functionality in biomedical applications [123].

By blending PLA with PGA (e.g., PLA/PGA mixtures), it is possible to modify the degradation rates, which can be tailored to suit the specific requirements of various implants. In the realm of ophthalmology, researchers have made significant strides in improving ocular implants by ingeniously combining PLA with polyvinylpyrrolidone (PVP). This innovative blend has revolutionized drug delivery systems within the eye, allowing for more precise control over medication release rates. Such advancements are particularly crucial in ophthalmic treatments, where the delicate nature of the eye demands exacting therapeutic dosages [124]. The physical properties of PLA are shown in Table 2.

In the realm of drug delivery, PLA plays a crucial role in creating sophisticated systems that can precisely control the release of medications within the body. This capability has revolutionized treatment approaches for numerous conditions, allowing for more targeted and efficient therapies. In orthopedic medicine, PLA has become a cornerstone material for devices that aid in bone healing and stabilization. Surgeons now rely on PLA-based screws and plates to provide robust support during the critical healing process of fractured or damaged bones [125].

In the specialized field of ophthalmology, PLA’s optical clarity makes it an excellent choice for intraocular lenses [126]. In orthopedics, PLA implants degrade over time, removing the need for additional surgeries. PLA is used in tissue scaffolds for regenerative medicine, supporting cell growth and tissue regeneration [125,127]. Its versatility also extends to controlled drug delivery systems, offering sustained drug release over time. PLA fibers have shown excellent cytocompatibility and biological safety, making them ideal for wearable devices [128].

Water vapor permeability is a key factor in evaluating the comfort and functionality of wearable devices. To assess this, the permeability of a flexible strain sensor was measured at room temperature and 30% relative humidity (RH). The sensor was tightly placed on a bottle containing 1.0 g of deionized (DI) water, and water vapor permeability was determined by tracking the remaining weight of the water [129]. For comparison, bottles were covered with pure PLA fibrous mats or cling film, or left uncovered. As concerns about electronic waste rise, there is a growing interest in biodegradable and bioabsorbable sensing materials [130]. A disposable sensor made from biodegradable flexible PLA piezoelectric film (DS-PLA) has been developed, offering significant sensitivity to both longitudinal compressive and transverse tensile forces [131]. This makes it suitable for use as a pressure or tensile sensor, expanding its potential applications in wearable and implantable electronics.

**Table 2:** Physical properties of PLA [120,132,133,134].

Property	Values
Specific gravity (g/cm^3^)	1–1.5
Surface energy (dynes)	35–40
Melting temperature (°C)	150–200
Molecular weight (gms)	2.7 × 10^−19^
Mass flow index (g/10 min)	5–22
Crystallinity (%)	5–40
Glass transition temperature (°C)	50–75
Solubility parameters (J/cm)	21
Mechanical flexibility	Low
Thermal conductivity (W/mK)	0.13–0.22
Electrical conductivity (S/m)	Non-conductive
Biocompatibility	Excellent

#### 2.2.2. Polycaprolactone (PCL)

Polycaprolactone (PCL) is a synthetic, biodegradable polyester with aliphatic properties, known for its mechanical strength, compatibility with various other polymers, and biodegradability. It gained notable attention during the resorbable-polymer boom of the 1970s and 1980s, especially in the fields of biomaterials and drug delivery. PPCL is a hydrophobic, slow-degrading synthetic polymer, making it ideal for long-term implantable devices and drug delivery systems [135]. Beyond these uses, PCL is also employed in food packaging and tissue engineering, broadening its applicability in both medical and commercial industries [136]. In the early 1990s and 2000s, interest in PCL resurged within the biomaterials field, driven by the rise of tissue engineering. PCL attracted attention due to its superior rheological and viscoelastic properties compared to other resorbable polymers, making it easier to process and shape into various scaffolds for tissue engineering applications [137].

PCL consists of hexanoate repeat units and is a semicrystalline polymer with a crystallinity degree of up to 69%. Its unit cell is orthorhombic, with lattice constants of a = 7.496 Å, b = 4.974 Å, and c = 17.297 Å, where c represents the fiber axis [138]. The structure of polycaprolactone (PCL) is made up of repeating caprolactone monomer units. The chemical formula for PCL is (C_6_H_10_O_2_)_n_, where “n” indicates the number of repeating units in the polymer chain. The monomer is a six-membered lactone ring with the formula C_6_H_10_O_2_ [139]. It contains a carbonyl group (C=O) and an oxygen atom that forms part of the ester link in the polymer backbone. The caprolactone monomers link together through a ring-opening polymerization process, forming long chains of polycaprolactone with ester linkages (-COO-) between the repeating units [140].

PCL is synthesized by ring-opening polymerization of ε-CL using metal alkoxides, metal carboxylates, or ionic initiators. While metal-based catalysts can be toxic and difficult to remove [141], calcium and magnesium-based catalysts are preferred due to their low toxicity and ability to produce high molecular weight. The resulting PCL is semicrystalline, with crystallinity affected by factors such as molecular weight, cooling rate, and impurities. PCL is a promising biomaterial because of its rubbery properties, adjustable biodegradability, and the ease with which it can be made into blends, composites, and copolymers [142,143].

In the realm of surgical sutures, PCL’s flexibility and strength come to the fore, ensuring wounds heal securely. Its role in drug delivery systems is equally impressive, offering a controlled release mechanism that can be fine-tuned to specific medical needs. One of PCL’s most remarkable features is its ability to have its surface roughness and hydrophilicity adjusted, allowing for optimal interaction with living tissues—a crucial factor in the success of medical implants [144]. Perhaps PCL’s most distinctive characteristic is its slow degradation rate. This property can be a game changer for long-term medical implants and tissue engineering projects. Depending on its molecular weight, PCL can persist in the body for months or even years, providing sustained support where needed.

This degradation timeline, typically spanning 2–3 years in biological environments, is influenced by various factors, including molecular weight, shape, and the presence of residual monomers [145,146]. The breakdown of PCL in the body is a fascinating process, with lipase enzymes playing a starring role in its enzymatic degradation. From a manufacturing perspective, PCL’s low melting point is a significant advantage, enabling various processing methods. This property has opened up exciting possibilities in the field of 3D printing, allowing for the creation of custom-designed implants and scaffolds with unprecedented precision [147,148]. Table 3 shows the physical and mechanical properties of PCL.

Self-healing properties are imperative for flexible devices in long-lasting wearable systems. A self-healing heater (SHH) was developed using electrospun PCL fibers, which have a melting point of 60 °C, eliminating the need for additional processes [151]. PCL is also used in capacitive pressure sensors, benefiting from its biodegradable nature and high sensitivity in electric double-layer (EDL) effect sensors [152]. Researchers have also implemented PCL as a polymer matrix for wearable radiation shielding materials due to its biocompatibility and electrospinning capability [153]. Furthermore, a functional membrane integrated into a microfluidic sampling interface and electrochemical sensing unit was developed using a PCL and polyethylene oxide (PEO) blend to prevent biofouling in wearable detection sensors [154].

Cardiac tissue engineering is advancing with the potential to improve heart function by regenerating contractile tissue, with PCL emerging as a key material for scaffolds that mimic the heart’s structure and mechanics [155,156,157]. PCL is also used in implantable drug delivery systems, such as a novel reservoir-type implant with a biodegradable membrane that controls drug release, using PCL and risperidone (RIS) as the model drug [158]. PCL and PCL–chitosan composite scaffolds loaded with procaine (PRC) were successfully fabricated through bioprinting for sustained local drug delivery [159]. PCL is also being blended with calcium phosphate composite nanostructures for effective antibacterial coatings on implants and medical devices [160].

#### 2.2.3. Polylactic-co-glycolic acid (PLGA)

Polylactic-co-glycolic acid (PLGA) is a synthetic biodegradable polymer with biocompatibility, non-toxicity, and versatility. Known as a “smart polymer”, it responds to environmental changes such as pH, temperature, and ionic strength [161]. PLGA is a copolymer of poly(lactic acid) (PLA) and poly(glycolic acid) (PGA), both used since the 1970s in applications such as absorbable sutures. PLGA’s balanced properties make it ideal for medical and pharmaceutical uses. The degradation rate of PLGA can be modified by adjusting the PLA/PGA ratio, enabling controlled drug release. PLGA has revolutionized the field of tissue engineering and biomedical applications. This versatile polymer serves as a temporary home for cells, encouraging them to grow and form new tissue while gradually breaking down into harmless byproducts that the body can easily eliminate [162,163].

PLGA has become an indispensable part in the world of surgery and surgical devices. It is used to create sutures that dissolve over time, eliminating the need for uncomfortable removal procedures. Wound dressings made from PLGA offer protection during healing and then disappear, reducing the risk of long-term complications or reactions. One of PLGA’s most promising features is its ability to act as a sophisticated drug delivery system [164]. Researchers are harnessing its degradation properties to create implants that can release medications in precise doses over extended periods. This innovation could transform patient care, potentially replacing frequent injections or pills with a single, long-lasting implant.

PLGA is composed of two distinct monomeric units: lactic acid and glycolic acid. These monomers undergo a polymerization process, forming a macromolecular structure where ester linkages (-COO-) serve as the connective tissue between the repeating units [165]. By adjusting the ratio of PLA to PGA units, polymer chemists can fine-tune critical properties such as the degradation kinetics and hydrophobicity of the resulting material. This level of control is crucial in biomedical applications, where the polymer’s behavior must be precisely matched to the physiological environment and the intended therapeutic purpose [166]. PLGA’s molecular backbone is the key to its biodegradability [167]. These bonds are susceptible to hydrolysis in biological systems, allowing the polymer to break down into its constituent monomers over time. The rate of this degradation is not fixed but can be modulated by altering the PLA/PGA ratio and manipulating processing conditions during polymer synthesis and device fabrication. PLGA’s molecular architecture can exhibit either an alternating or a random distribution of PLA and PGA units along the polymer chain. This structural variability contributes to the polymer’s versatility, allowing for a spectrum of properties that can be tailored to specific biomedical applications.

The flexibility of PLGA extends beyond its molecular structure to its macroscopic properties. It can be processed into a diverse array of physical forms, from nanoparticles to scaffolds, and has the capacity to encapsulate molecules of varying sizes. PLGA exhibits remarkable solubility across a spectrum of organic solvents, ranging from halogenated compounds to cyclic ethers, ketones, and esters, facilitating diverse processing methods and formulation strategies in biomedical applications [168]. In water, PLGA biodegrades via hydrolysis of its ester linkages. PLA’s methyl side groups make it more hydrophobic than PGA, leading to slower degradation in lactide-rich PLGA copolymers, which absorb less water [169]. The degradation of PLGA brings about changes in properties such as glass transition temperature, moisture content, and molecular weight, influencing drug release rates.

PLGA is synthesized through random melt co-polymerization of lactide and glycolide under a high vacuum using a catalyst like stannous octoate, with the reaction maintained at 160–190 °C [170]. Lactic acid and glycolic acid, derived from fermentation or chemical processes, are used to obtain the lactide and glycolide dimers. PLGA is purified by dissolving in chloroform, precipitating in ethanol, and drying in a vacuum. It is available with ester or acid end groups. The biocompatibility, biodegradability, and adaptive features of PLGA have garnered significant interest across several disciplines [171]. However, its fast degradation can lead to the accumulation of acidic byproducts, which may cause inflammation, tissue damage, and instability in encapsulated drugs. It is crucial to optimize PLGA’s degradation rate and pH to ensure its safety and effectiveness in specific applications [172].

The degradation and drug release rate of PLGA can be accelerated by increasing its hydrophilicity, enhancing chemical interactions among hydrolytic groups, reducing crystallinity, and increasing the device’s volume-to-surface ratio of the device, which are critical factors influencing its degradation behavior [173]. By carefully managing these parameters, researchers can optimize PLGA’s performance for specific biomedical applications, ensuring controlled and sustained drug release. A deep understanding of PLGA’s characteristics and degradation mechanisms is essential for designing effective drug delivery systems and biomedical devices that provide reliable, long-term therapeutic benefits [174]. The material’s excellent processability further enhances its utility, enabling the fabrication of implants with complex geometries tailored to meet diverse clinical needs. This versatility positions PLGA as a promising material for advancing patient care through innovative treatment options and improved medical outcomes [175]. Table 4 shows the physiochemical properties of different PLGA materials.

PLGA is commonly used in both wearable and implantable devices due to its versatility and ability to be tailored for controlled drug release. Smart wearable patch systems combining biosensing and therapeutic components are emerging as promising solutions for personalized healthcare [176]. These systems offer noninvasive, user-friendly, and long-acting smart drug delivery. For example, insulin-loaded PLGA nanoparticles in alginate microgel depots release drugs when subjected to stretching and bending forces [177]. In wearable devices, PLGA has been used for sustained drug release, such as in a methafilcon contact lens with anti-glaucoma drug latanoprost, providing up to 4 weeks of release without ocular toxicity [178,179]. Additionally, ultrasound-responsive PLGA microparticles have been developed for cancer treatment, with flexible, wearable patches that use ultrasonic energy for controlled drug delivery [180].

PLGA is also used in implantable devices like tissue scaffolds, drug delivery systems, and biosensors. Micro-molding and electrohydrodynamic (EHD) printing techniques enable the creation of precise, implantable PLGA devices [181]. PLGA implants have been used to deliver proteins like ovalbumin, lysozyme, and bovine serum albumin. In one study, PLGA incorporated with ciprofloxacin was deposited on titanium plates using the MAPLE technique, showing antibacterial properties and prolonged drug release for up to 45 days, making it promising for treating bone infections [182,183]. In wearable devices, PLGA has been used for sustained drug release, such as in a methafilcon contact lens with anti-glaucoma drug latanoprost, providing up to 4 weeks of release without ocular toxicity. Additionally, ultrasound-responsive PLGA microparticles have been developed for cancer treatment, with flexible, wearable patches that use ultrasonic energy for controlled drug delivery. Additionally, PLGA-functionalized scaffolds, like those for breast cancer targeting with gefitinib, highlight its versatility and potential for various therapeutic applications [184].

**Table 4:** Physiochemical properties of different PLGA materials [185,186,187,188].

Polymer	Modulus (GPa)	Elongation (%)	Mechanical Flexibility	Thermal Conductivity (W/mK)	Electrical Conductivity (S/m)	Biocompatibility	Crystallinity (%)	Degradation Time (Weeks)
Polyglycolide/ polyglactin	7	15–20	Low	0.2–0.4	Non-conductive	Excellent	45–55	6–12
Poly(L-lactide)	2.7	5–15	Moderate	0.2–0.4	Non-conductive	Excellent	37	12–18
Poly(D, L-lactide)	2.9	3–10	Moderate	0.2–0.4	Non-conductive	Excellent	Amorphous	11–15
Poly(D, L-lactide-co-glycolide) 85/15	2	3–10	Moderate	0.2–0.4	Non-conductive	Excellent	Amorphous	5–6
Poly(D, L-lactide-co-glycolide) 75/25	2	3–10	Moderate	0.2–0.4	Non-conductive	Excellent	Amorphous	4–5
Poly(D, L-lactide-co-glycolide) 50/50	2	3–10	Moderate	0.2–0.4	Non-conductive	Excellent	Amorphous	1–2

#### 2.2.4. Polyhydroxybutyrate (PHB) and Polyhydroxyvalerate (PHV)

Polyhydroxyalkanoates (PHAs) are a diverse class of naturally occurring, biodegradable, and biocompatible polyesters produced by various microorganisms. These polymers serve as intracellular energy storage in bacteria, much like how humans store fat, and have gained significant attention for their potential applications in a range of industries, including medicine, pharmacology, and environmental sustainability [189]. PHAs, especially polyhydroxybutyrate (PHB) and polyhydroxyvalerate (PHV), are gaining attention for their potential to be produced from renewable resources, positioning them as promising alternatives to traditional plastics [190]. PHB is a rigid, crystalline polymer that is biodegradable and has applications in packaging, medical devices, and agriculture. However, its brittleness limits its use in some applications. In contrast, PHV, a copolymer of PHB, contains valerate (C_5_) units in its polymer chain, which enhances its flexibility and reduces crystallinity compared to PHB [191].

Both PHB and PHV are biodegradable, meaning they naturally break down in the environment. This presents a promising solution to the growing plastic pollution problem. However, the production of these polymers is more complex and expensive than that of traditional plastics due to the need for specific carbon sources and the biotechnological processes involved in their synthesis [192]. Ongoing research efforts are dedicated to improving production techniques and reducing the costs associated with PHAs, a development that could significantly expand their application across various industries. Among the PHAs, PHB and PHV stand out for their exceptional potential as bioplastics. Their unique combination of biodegradability, biocompatibility, and sustainable production from renewable resources positions them as promising materials for a wide range of applications, including medicine, packaging, and environmentally friendly solutions [193].

PHB is composed of repeating 3-hydroxybutyrate units, forming a molecular architecture that resembles a chain with regularly spaced links. Its carbon backbone, adorned with ester linkages, creates a highly organized structure. The repeating unit, C_4_H_8_O_2_, represents the 3-hydroxybutyrate monomer, which can be visualized as the building block of this molecular chain [194]. The highly crystalline nature of PHB resulting from this ordered arrangement is responsible for its characteristic rigidity and brittleness. Delving deeper into PHB’s structure, we find that each repeating unit consists of [-CH_2_-CH(CH_3_)-COO-]. This sequence forms the polymer chain through ester bonds, where the carbonyl group (C=O) of one unit connects to the hydroxyl group (-OH) of the next, creating a continuous, interlocked molecular structure [195]. PHV, on the other hand, showcases the versatility of these biopolymers. As a copolymer, it combines two distinct monomers: the 3-hydroxybutyrate from PHB and 3-hydroxyvalerate, which is derived from valeric acid (C_5_H_10_O_2_) [196].

The incorporation of the valerate unit (C_5_) into the polymer chain brings about a significant transformation in the material’s properties, reducing its crystallinity and enhancing its flexibility compared to PHB. The repeating units of PHV alternate between 3-hydroxybutyrate (C_4_) and 3-hydroxyvalerate (C_5_). The general structure of PHV is [-CH_2_-CH(CH_3_)-COO-] for the 3-hydroxybutyrate unit [-CH_2_-CH_2_-CH_2_-CH_2_-COO-] [197]. This unique arrangement results in a polymer chain that maintains the ester linkages characteristic of PHB but introduces a more flexible structure due to the presence of the valerate units.

Species such as *Ralstonia eutropha*, *Azotobacter*, and various *Bacillus* strains have evolved the ability to produce PHB and PHV as a means of energy storage. When these microorganisms are provided with an abundance of carbon-rich substrates, such as glucose, sucrose, or fatty acids, they begin to accumulate PHB. However, this accumulation is triggered specifically under conditions of nutritional stress, such as limited nitrogen or phosphorus availability. In essence, these microorganisms convert excess carbon into a stored form of energy, much like how humans and other animals store excess energy as fat [198].

The carbon is converted into acetyl-CoA, which is a precursor for PHB production. The acetyl-CoA then undergoes polymerization. After fermentation, PHB is extracted from the microbial cells using solvents like chloroform or acetone and purified. PHV is produced similarly to PHB, but with the incorporation of valerate units (C_5_) [199]. This process uses a mixture of carbon sources, including glucose and valeric acid (or its precursors, like sodium valerate), which provide the C_5_ units needed for PHV. The microorganism metabolizes the valeric acid into 3-hydroxyvalerate (C_5_ monomer), which is polymerized into PHV. Like PHB, PHV is extracted using solvents and purified [200,201]. PHV can also be produced as a copolymer with PHB, called polyhydroxybutyrate-co-valerate (PHBV), combining both 3-hydroxybutyrate and 3-hydroxyvalerate units in the polymer chain.

PHB and PHV are biodegradable and biocompatible polymers, making them highly suitable for biomedical applications. Since PHB and PHV can degrade naturally in the body, it minimizes the need for surgical removal, making them ideal for uses such as sutures, drug delivery systems, and tissue scaffolds [202]. Both polymers are non-toxic to living tissue, minimizing immune responses due to their natural origin and feasibility to human cellular components. PHB is rigid and has high tensile strength but is brittle, which limits its flexibility. In contrast, PHV is more flexible and pliable, making it suitable for dynamic applications like flexible wound dressings and vascular grafts [203].

Blending PHB and PHV can balance rigidity and flexibility, optimizing their mechanical properties for various biomedical uses. Table 5 shows the physical and mechanical properties of both PHB and PHV. The degradation rate of PHB is slower but can be tailored, while PHV degrades more quickly due to its increased flexibility. This controlled degradation is advantageous in tissue engineering, where the material degrades at a rate that matches tissue growth [204].

Both PHB and PHV are ideal for use in scaffolds, where they degrade over time, allowing the body to replace the material with its own cells. PHAs derived from Gram-negative organisms face challenges in biomedical applications due to contamination with lipopolysaccharide endotoxins, which can trigger immune reactions and limit their use in medical implants [208,209]. To address this issue, PHB reinforced with chitosan (CS) has been developed into piezoelectric films, improving its mechanical and electrical properties, making it suitable for large-scale production of implantable bioelectronic devices [210]. To enhance the lifespan of implantable sensors, these components can be encapsulated in durable polymers like PHB and poly(octamethylene maleate (anhydride) citrate) (POMaC), which resist degradation in bodily fluids, extending the sensors’ operational lifespan [211].

PHB itself has inherent piezoelectric properties, stemming from its crystalline phases, particularly the orthorhombic α-phase and electroactive β-phase, allowing it to generate electrical charges under mechanical stress [212]. These properties make PHB a promising material for implantable piezoelectric scaffolds, which can stimulate cellular activity, promote tissue regeneration, and provide therapeutic benefits in biomedical applications [213]. Advances in PHB–chitosan films and polymer encapsulation open new possibilities for PHB in bioelectronics and tissue engineering, despite challenges with endotoxin contamination [214].

#### 2.2.5. Polydioxanone (PDO)

Polydioxanone (PDO), also known as polydioxanone suture (PDS), is a biodegradable polymer. It has been broadly utilized in biomedical applications due to its wide range of properties. PDO is synthesized via ring-opening polymerization of p-dioxanone in the presence of an organometallic catalyst and thermal energy. Its major characteristics include its nonantigenic and nonpyrogenic biocompatibility, which results in minimal tissue reaction during absorption after implantation. Such a property is important for minimizing the immune responses, making PDO suitable for a variety of biomedical devices [215].

Physically, PDO is semi-crystalline, with a crystallinity of about 55%, a glass transition temperature of between −10 °C and 0 °C, and a melting temperature of around 110–115 °C [215]. Such properties allow PDO to be processed excellently at low temperatures, avoiding spontaneous depolymerization during fabrication. PDO also has excellent mechanical properties, including high elasticity, which is particularly advantageous for tissue repair applications, such as soft tissue regeneration [216]. PDO’s shape memory property offers rebound and kink resistance, which makes it suitable for use in vascular conduits, although it may pose challenges in knot retention when used as sutures [217].

The degradation process of PDO results in products that are metabolically compatible with the body, with a degradation rate that typically lasts 6–8 months. This intermediate absorption time is advantageous for tissue engineering applications, as it provides the material to maintain mechanical stability during the healing process [216]. The slow hydrolytic degradation mechanism ensures that PDO is fully metabolized in the body, with metabolites being excreted by the kidneys or exhaled as carbon dioxide [218,219].

In surgical applications, PDO sutures exhibit great drift characteristics, which makes them easy to glide through tissues. PDO retains more packaging memory than counterparts like poliglecaprone 25 and glycomer 631, which alters its flexibility and handling. However, it is always a trustworthy choice for surgeries requiring longer absorption times. For example, in procedures like the Nuss procedure for pectus excavatum, PDS showed safety and effectiveness, which offers advantages like reduced operation duration, lower blood loss, and decreased postoperative pain compared to traditional materials like surgical steel wires [220]. The absorption time of PDO is ~6 months, with remnants still detectable after this period. Due to this, PDO is a favorable option for surgical applications where temporary support is needed without long-term foreign material presence [221].

The mechanical properties of PDO closely match the major structural components of native extracellular matrix (ECM), such as collagen and elastin, which makes it suitable for tissue engineering. Its low melting leads it to being processed using centrifugal spinning techniques, which is particularly useful for creating scaffolds for soft tissue repair [216]. PDO’s significant water absorption capacity further extends its applicability in biomedical applications, with swelling rates of approximately 43.6% for membranes of 0.25 mm thickness within 24 h [218].

### 2.3. Natural Polymers

#### 2.3.1. Polysaccharides

Polysaccharides are complex natural polymers composed of long chains of monosaccharide units, typically containing anywhere from 20 to 60,000 monosaccharide molecules. These macromolecules are fundamental in various biological processes and serve important roles in nature [222]. The study of their chemistry covers several key areas, including their natural occurrence, classification, and structural components. Understanding the primary structure of polysaccharides is critical, and this is determined using a variety of structural analysis methods, such as spectroscopy, chromatography, and enzymatic degradation [223].

Polysaccharides’ significance extends beyond their basic biological functions, especially in medicinal and biomedical fields. They have applications ranging from drug delivery systems to wound healing, making their study crucial in the development of therapeutic strategies [224]. To enhance or modify the properties of polysaccharides for these applications, molecular modifications are often required. These modifications can be achieved through chemical, physical, or biological approaches, each with distinct advantages and challenges.

Alginate, a naturally occurring polysaccharide, was first described by British chemist E.C.C. Stanford in 1881 and is primarily derived from brown seaweed. In 2019, global seaweed production reached 34.5 million tons, with brown and red seaweed making up 99% of the total, while green seaweed contributed only 0.04% [225]. Brown seaweed, which is a renewable biomass, is abundant worldwide and used in eco-friendly bio-production processes. Alginate, present in up to 40% of brown seaweed dry weight, is a hydrophilic anionic polysaccharide. It can be derived from both seaweed (Phaeophyceae) and bacteria.

The alginate extracted from brown seaweed is often in the form of sodium, magnesium, calcium, or other metal salts and can be transformed into various metal alginate gels, such as those with calcium, barium, copper, or zinc [226]. Alginic acid is a natural copolymer made up of long chains of two acidic polysaccharides: α-L-guluronic acid (G block) and β-D-mannuronic acid (M block), with alternating MG blocks as well. These units are connected by 1,4-glycosidic linkages [227]. Each uronic acid ring contains a carboxylic acid (COOH) and two hydroxyl (OH) groups.

The arrangement of G and M blocks, and their chemical composition, varies between seaweed species, different parts of the algae, and the harvest season. The strength and stiffness of alginate gels depend on the quantity and length of G blocks within the alginate chains [228]. Alginate has various medical applications due to its unique properties, such as biocompatibility, biodegradability, and the ability to form gels. It finds its applications in drug delivery systems, tissue engineering, dental applications, and biosensors [229,230].

Chitosan is classified as a mucopolysaccharide, typically containing both glucosamine and acetylglucosamine residues [231]. It is considered chitosan when the acetylglucosamine concentration is below 50%, and chitin when it is 50% or higher. Chitin, second only to cellulose in abundance, is a key source of carbon and nitrogen in marine ecosystems [232]. Chitosan is a linear polymer made of D-glucosamine and N-acetyl-D-glucosamine subunits linked by 1,4-glycosidic bonds.

Its structure includes functional groups like amino and hydroxyl groups, which influence its solubility and mechanical properties [233]. Chitosan is more soluble than chitin in acidic conditions, primarily due to the protonation of its amino group, which transforms it into a polyelectrolyte. This solubility makes chitosan useful in various fields such as agriculture, medicine, and industry [234]. It can be extracted directly from fungi or produced by deacetylating chitin. Fungi typically produce more chitin than chitosan, and chitosan is primarily found in zygomycetes. Extraction from fungi is advantageous, as it can be carried out year-round. Another major source of chitin is the exoskeletons of marine crustaceans, especially from the shrimp industry [235].

Chitosan is the only natural polycation, and its solubility depends on its acetylation degree, molecular weight, and pH. Chitosan oligomers are soluble across a broad pH range, but higher-molecular-weight chitosan is only soluble in acidic media, limiting its use in some physiological applications [236]. This has led to the development of chitosan derivatives with improved solubility. Chitosan has several medical applications due to its biocompatibility, including wound healing, tissue engineering, drug delivery systems, and cancer therapy [237,238].

In 1934, Karl Meyer and John Palmer discovered hyaluronic acid, a substance containing two sugar molecules, one of which was uronic acid, isolated from the vitreous body of cows’ eyes [239]. Hyaluronan, the salt form of hyaluronic acid, is an essential component of extracellular matrices (ECMs) in vertebrate tissues. It is found in the vitreous humor of the eye, synovial joint fluid, the matrix around oocytes before ovulation, and pathological matrices like those in coronary restenosis [240]. The largest amount of hyaluronan (around 7–8 gms in an average adult human) is in the skin, where it is present in both the dermis and the epidermis. While the dermis contains less hyaluronan, the epidermis has a higher concentration of hyaluronan in the matrix around the cells, estimated at 2–4 mg/mL [241].

Hyaluronic acid is a naturally occurring linear polysaccharide composed of repeating disaccharide units of N-acetyl-D-glucosamine and D-glucuronic acid, linked by β (1,4) and β (1,3) glycosidic bonds. Its molecular weight ranges from 10^4^ to 10^7^ Da [242]. Hyaluronic acid is unique due to the absence of sulfated groups and covalently linked peptides. It plays a critical role in synovial fluid (SF) in joints, acting as a lubricant and regulating viscosity. Healthy joints contain around 2.26 g/L of hyaluronic acid. The polysaccharide chains are linear, unbranched, and coil into a conformation.

In tissues, HA’s molecular weight varies, with normal tissues having a hyaluronic acid molecule of 10 MDa, which is approximately 25 mm long and 1 nm thick [243]. In the biomatrix, hyaluronic acid typically has a molecular weight of 6–12 MDa, with healthy joints having an average molecular weight of around 7 MDa and unhealthy joints 4.8 MDa. Hyaluronic acid (HA) has a wide range of medical applications due to its natural presence in the body and its unique properties, such as its ability to retain moisture and facilitate tissue repair [244]. Hyaluronic acid is mostly studied for its potential in osteoarthritis treatment, cell therapy, cataract surgery, dry eye treatment, and other regenerative medicine applications due to its ability to support tissue regeneration [245,246].

In 1838, French chemist Anselme Payen discovered cellulose while studying wood, isolating it from plant matter and identifying it as a complex carbohydrate made of glucose molecules [247]. Named for its origin in plant cell walls, cellulose is the most abundant natural polysaccharide on Earth, providing structural support to plants [248]. Though indigestible by humans, it is an important part of dietary fiber. Cellulose is made up of long, unbranched chains of β-D-glucose molecules linked by β-1,4-glycosidic bonds, giving it a linear and rigid structure.

The chains form strong hydrogen bonds both within and between chains, contributing to its high tensile strength and insolubility. Multiple cellulose chains bundle into microfibrils, which are key structural components of plant cell walls [249]. Cellulose has a semi-crystalline structure with alternating crystalline and amorphous regions, where crystalline regions provide strength and amorphous regions allow for flexibility. Cellulose is important in medical applications for several reasons, mainly due to its unique properties, such as biocompatibility and biodegradability, and it finds its use in surgical sutures, medical coatings, and wound dressings as hydrogels [250,251].

#### 2.3.2. Proteins

Proteins are complex molecules and one of the basic types of natural polymers that play a critical role in the structure, function, and regulation of the cells, tissues, and organs in living organisms. They are made up of chains of smaller units called amino acids, which are linked together by peptide bonds [252]. The sequence of amino acids in a protein determines its unique 3D shape and specific function within the body. There are twenty different amino acids that combine in various ways to form proteins [253]. These amino acids are categorized as essential and non-essential. The primary structure of protein consists of a linear sequence of amino acids, and the secondary structure comprises local folding of the polypeptide chain into structures like alpha helices or beta sheets. The tertiary structure is the overall 3D shape of the protein formed by the folding of secondary structures. The quaternary structure is the result of arranging multiple protein subunits [254]. A rapidly progressing area of protein engineering involves the design of sequences rather than the modification of existing natural proteins. A rapidly advancing field in protein engineering focuses on designing sequences rather than modifying existing natural proteins. Although this approach is considerably more challenging, it is a crucial effort, as it enhances our understanding of protein function [255].

Collagens are the most abundant fibrous proteins in both soft and hard tissues, such as tendons, skin, bones, and teeth, accounting for about 30% of total protein in mammals. These proteins not only serve as structural components of tissues but also form bio-nanocomposites with minerals, particularly in hard tissues [256]. There are twenty-nine types of collagens, classified by their structure and function, including fibril-forming, network-forming, and transmembrane collagens. Collagen consists of three polypeptide chains that form a triple helix, creating a stable, rod-like structure. These triple-helical molecules aggregate to form fibrils, which can vary in diameter depending on the tissue [257].

Collagen fibrils are further organized into fibers in tissues like tendons and ligaments. The structural arrangement provides strength and support to tissues, with variations in structure and composition across different collagen types. Type I collagen, the most common, is found in tendons, skin, and bones, while type II is mainly found in cartilage and type III in extensible tissues like skin and blood vessel walls [258]. In bone, collagen fibrils incorporate hydroxyapatite crystals to enhance stiffness, while in tendons and ligaments, multiple fibrils combine to form collagen fibers. Collagen has several important medical applications, including scaffolds for tissue repair, dressings for wound healing, cosmetic surgeries, and dental applications [259].

Gelatin is another natural protein polymer derived from the hydrolytic degradation of collagen [260]. Gelatin is commonly extracted through boiling or hydrolysis, producing a flavorless and colorless substance used primarily as a gelling agent in food production. It contains 19 amino acids, with glycine, proline, and hydroxyproline being the predominant ones [261]. The primary sources of gelatin are pig skin, bovine hides, and bones, with pig skin contributing the most to global production. Alternative sources, including fish, birds, and other animals, are also explored.

Gelatin is composed of 18 types of complex amino acids, with glycine, proline, and hydroxyproline making up 57% of its structure [262]. The remaining 43% consists of amino acids like glutamic acid, alanine, and aspartic acid. Chemically, gelatin contains 25.2% oxygen, 6.8% hydrogen, 50.5% carbon, and 17% nitrogen. Its structure includes various polypeptide chains, such as α-chains, β-chains, and γ-chains, with molar masses of approximately 90 × 10^3^, 180 × 10^3^, and 300 × 10^3^ g/mol, respectively. When heated, gelatin dissolves into colloids but forms a gel at temperatures below 35–40 °C [263].

Prolonged boiling alters its properties, preventing reformation upon cooling. The viscosity and gel strength of gelatin are influenced by factors like molecular mass distribution, electrolyte concentration, pH, and temperature. Its distinctive structure of amino acids gives it several medical benefits. Generally, gelatin comes in the form of tablets, granules, or powders, and sometimes it can be dissolved in water before use. Gelatin is widely explored by researchers as a matrix for three-dimensional cell culture and as a component of tissue-engineering scaffolds [264].

Silk fibroin, a versatile protein polymer primarily derived from *Bombyx mori* silkworms and other arachnids, has a long history of use in textiles and surgical sutures. Silk fibroin consists of a light (L) chain and a heavy (H) chain linked by a disulfide bond, forming a complex that interacts with glycoprotein P25 to create a micellar unit for fibroin transport [265]. The H-chain is responsible for the material’s strength, forming β-sheet crystallites, while the L-chain plays a lesser role. The H-chain contains a high proportion of glycine, alanine, and serine, and is structured with alternating hydrophobic and hydrophilic domains, contributing to the formation of crystalline and semi-amorphous regions [266].

Proline residues aid in chain folding during silk production, giving silk fibroin its unique mechanical properties. Its remarkable properties, such as strength, biocompatibility, and biodegradability, make it highly versatile for a variety of emerging biomedical applications [267]. The ability to process silk fibroin into multiple forms, like fibers, films, and hydrogels, adds to its appeal in diverse fields, including tissue engineering, drug delivery, and environmental sustainability [268].

Fibrinogen is a 340 kDa glycoprotein found in human blood plasma. Present at concentrations of 1.5–4 g/L, fibrinogen is a soluble protein that converts into an insoluble clot or gel when acted upon by the enzyme thrombin [269]. This conversion is triggered by a cascade of enzymatic reactions following vessel injury, activated blood cells, or a foreign surface [270]. Fibrinogen’s α-helical coiled-coil structure was initially classified alongside other fibrous proteins, like keratin and myosin. Fibrinogen consists of three pairs of polypeptide chains, Aα, Bβ, and γ, with molecular masses of 66,500, 52,000, and 46,500 Da, respectively. Post-translational modifications add carbohydrates to the Bβ and γ chains, bringing the total mass to about 340 kDa [271].

The polypeptide composition is denoted as (Aα Bβ γ)2, with fibrinopeptides A and B cleaved by thrombin to form the α and β chains. The chains are held together by 29 disulfide bonds, with unique Cys-Pro-X-X-Cys sequences forming disulfide rings. Interchain disulfide bonds link the two halves of the molecule, stabilizing its structure [272]. Fibrinogen is widely used in medical applications due to its essential role in blood clotting and wound healing. Some of the key medical uses include hemostasis and bleeding control, wound healing, surgeries, and drug delivery systems [273,274].

### 2.4. Emerging Polymers

#### 2.4.1. Polyphosphazenes (PPZs)

Polyphosphazenes (PPZs) are hybrid inorganic–organic polymers that have attracted significant attention for their utilization in wearable and implantable biomedical devices. Their molecular structure consists of alternating phosphorus and nitrogen atoms with organic side groups attached to each phosphorus center. This will provide a great degree of structural flexibility and enable better control over their physicochemical and biological properties [275,276]. Such tunability enables PPZs to be modified for specific applications by adjusting their hydrophilicity, hydrophobicity, degradability, and mechanical strength, making them easy to integrate in biomedical devices.

PPZs are biocompatible and biodegradable, which makes them ideal for biomedical applications. PPZs are unlike many other biodegradable polymers that degrade into acidic byproducts, which can lead to localized pH drops and inflammatory responses. Rather, it hydrolyzes into non-toxic degradation products such as phosphate and ammonia, which help in maintaining a stable physiological environment [277]. The degradation rate of PPZs can be managed by modifying the side groups, allowing for tailored degradation profiles suitable for drug delivery or implantable devices that require gradual breakdown over months or years [278].

PPZs also have tunable mechanical properties, as a result of which they can form rubber-like elastomers with a glass transition temperature as low as −100 °C to rigid, brittle materials [279]. Such a wide range of mechanical adaptability is beneficial for wearable devices, where flexibility and durability are crucial, as well as for implantable devices that require materials with specific mechanical strengths to integrate in biological tissues [278,279]. Their inherent resistance to water, solvents, oils, and chemicals, and their natural flame retardancy and thermal stability, makes them suitable for use in various physiological environments [279].

Another feature of PPZs is their ability to form nanoparticles and hydrogels, which are essential for advanced drug delivery systems. These materials are capable of encapsulating therapeutic molecules, protecting them from premature degradation while ensuring controlled release at the target site, and thereby improving treatment efficacy and minimizing side effects [275]. Such capability is advantageous in wearable drug delivery patches and implantable reservoirs that are designed for sustained drug release over extended periods.

PPZs exhibit a remarkable versatility that extends beyond their structural adaptability, venturing into the realm of advanced biomedical imaging. The ability to incorporate functional groups such as fluorophores into their molecular architecture opens up exciting possibilities for bioimaging applications. This innovative feature allows for real-time, non-invasive monitoring of implanted devices or drug delivery systems within the living body. Clinicians can now observe the function and degradation of these PPZ-based systems without resorting to invasive procedures, providing valuable insights into their performance and longevity [277].

The fusion of imaging capabilities with therapeutic applications represents a significant advancement in the field of precision medicine. PPZ-based systems can potentially serve dual roles, simultaneously delivering targeted treatments and providing visual feedback on their efficacy and distribution within the body. The unique combination of inorganic and organic components in PPZs, coupled with their structural adaptability, controlled degradability, biocompatibility, and multifunctionality, positions them as an exceptional class of materials for the development of next-generation wearable and implantable biomedical devices [278].

#### 2.4.2. Sundew-Inspired Adhesive Hydrogels

Sundew-inspired adhesive hydrogels represent a fascinating frontier in biomaterials research, particularly for biomedical applications. These innovative materials draw inspiration from the remarkable adhesive properties of the sundew plant, known for its ability to capture insects using a sticky mucilage secreted by its leaves. By mimicking this natural adhesive mechanism, researchers have developed synthetic hydrogels that offer a unique combination of strong adhesion, self-healing capabilities, and biocompatibility.

The composition of these hydrogels primarily consists of boronic acid-conjugated alginate (AlBA) and tannic acid (TA), a polyphenol commonly found in plants. The interaction between these components through reversible boronic acid-cis-diol complexation results in a viscoelastic gel that swells under physiological conditions. This swelling behavior contributes to the hydrogel’s strong adhesive properties, which are crucial for effective tissue sealing and potentially reducing the need for traditional suturing techniques [280].

One of the most intriguing aspects of these hydrogels is their pH-responsive mechanical strength. The tensile strength of the hydrogel-based threads is notably higher in acidic environments, making them particularly suitable for applications in areas such as the stomach and intestines. This adaptability to different pH conditions broadens the potential applications of these materials in various physiological contexts. The incorporation of tannic acid into these hydrogels enhances their functionality beyond mere adhesion. Tannic acid imparts antioxidant, antibacterial, and hemostatic properties to the hydrogels, making them particularly valuable for wound healing and cell therapy applications.

Another significant advantage of sundew-inspired hydrogels is their ability to provide controlled release of tannic acid over time while maintaining their mechanical integrity during degradation. This feature ensures sustained therapeutic effects and consistent performance throughout the healing process [281]. The potential of these hydrogels extends beyond serving as alternatives to conventional sutures. Their adhesive properties and biocompatibility make them excellent candidates for various bioadhesion-based applications, such as advanced wound dressings. In these applications, the hydrogels can promote faster healing and tissue regeneration by providing a supportive environment for cellular processes. Perhaps one of the most promising aspects of these hydrogels is their ability to mimic the extracellular matrix environment. This characteristic supports cell proliferation and attachment, particularly for fibroblast-like cells, which are crucial for tissue regeneration [282].

The incorporation of nanomaterials, such as nanofibers and nanoparticles, into the hydrogel matrix represents a significant leap forward in biomaterial engineering. This innovative approach enhances the hydrogel’s mechanical strength and its ability to respond to environmental changes, much like the sundew plant’s remarkable adaptability to its surroundings [282]. These bio-inspired hydrogels have been explored for wearable and implantable devices due to their flexibility, strong adhesion, and bioactive properties.

In wearable sensors, these hydrogels provide high water content and excellent adhesion, which are important for keeping contact with the skin and reliable performance over time. Their self-healing properties, combined with the integration of nanoparticles, increase their durability and functionality in dynamic environments. As a result, it makes them suitable for long-term use in medical devices. Additionally, their property to regulate physical and chemical interactions, such as electrostatic and chemical bonds, makes them suitable for implantable devices. It is because biocompatibility and strong adhesion are important for interfacing with tissues and organs [283].

These hydrogels have exhibited great results in improving wound rehabilitation rates compared to untreated groups. This is due to their ability to facilitate cell adhesion and transfer wound healing factors, which is important for wound closure and healing [283]. The use of calcium ions (Ca^2+^) in the hydrogel matrix not only aids in the gelation process but also modulates keratinocyte proliferation and differentiation, promoting epidermal regeneration and dermal reconstruction during wound healing [284]. The development of these bio-inspired hydrogels represents a great opportunity in biomaterials. The convergence of nanotechnology and biomimetics create more efficient, responsive, and biocompatible materials for various applications like tissue engineering, wound healing, and medical device technologies.

#### 2.4.3. MXenes

MXenes are two-dimensional (2D) transition metal carbides, nitrides, and carbonitrides with the general formula M_n+1_X_n_T_x_ (where M represents transition metals like Ti, V, and Nb; X is carbon and/or nitrogen; and T_x_ denotes surface-terminating groups such as −OH, −F, and −O). These show a wide range of properties that make them suited for BioMEMS applications. These materials have ultrathin atomic layers and a high surface area, which provides them with exceptional mechanical toughness. The presence of surface functional groups, like hydroxyl (-OH), fluorine (-F), and oxygen (-O), makes MXenes hydrophilic. Due to this, their interactions with biological molecules are improved, enhancing their biocompatibility [285,286]. Their hydrophilicity makes them suitable for application in drug loading and delivery, and hence makes them ideal candidates for biomedical implants and sensors [287].

MXenes have captured the attention of researchers in the biomedical field due to their exceptional properties. Their electrical conductivity is particularly impressive, with Ti_3_C_2_T_x_ thin films demonstrating values exceeding 20,000 S/cm. This outstanding conductivity, coupled with their mechanical stability, positions MXenes as ideal candidates for BioMEMS and wearable sensors that require efficient charge transfer [287]. The optical properties of MXenes further enhance their appeal for biomedical applications. They exhibit strong broadband absorption and excellent light-to-heat conversion capabilities, making them particularly suitable for photothermal therapy (PTT) and photoacoustic (PA) imaging [286]. Their high photothermal conversion efficiency is especially valuable for targeted cancer treatments using PTT [288,289].

One of the most intriguing aspects of MXenes is their compositional versatility. By tailoring their composition, researchers can optimize these materials for specific biomedical applications. For instance, MXenes based on high-atomic-number metals like tantalum (Ta) and tungsten (W) offer strong X-ray attenuation, making them excellent candidates for contrast agents in computed tomography (CT) imaging [288]. The mechanical properties of MXenes are equally impressive. Materials such as Ti_3_C_2_T_x_ and Nb_4_C_3_T_x_ demonstrate remarkable stiffness, with values ranging from 300 to 400 GPa. This mechanical robustness makes them well suited for high-performance bioelectronic devices that must withstand various stresses in biological environments [287]. Furthermore, the magnetic and semiconducting characteristics of certain MXenes open up additional avenues in biomedical applications, particularly in drug delivery and imaging [288].

The surface chemistry of MXenes offers a remarkable degree of flexibility. This adaptability allows researchers to modify the surface of MXenes with precision, opening up a world of possibilities for enhancing their functionality. This ability to tailor MXenes at the molecular level is particularly valuable in the field of BioMEMS. It allows for the creation of highly specialized devices that can perform complex functions within biological environments [285]. Overall, the combination of excellent electrical conductivity, mechanical stability, optical properties, biocompatibility, and tunable surface functionalization makes MXenes a versatile material for the next generation of biomedical applications [287,289].

#### 2.4.4. Piezoelectric Biomolecular Materials and Transient BioMEMSs

Piezoelectric biomolecular materials and transient BioMEMSs represent a sophisticated intersection of bioengineering and materials science [290]. Composed of naturally derived components such as peptides, proteins, and other biological macromolecules, these materials have the unique ability to generate electrical signals in response to mechanical stress. This property makes them highly valuable for developing self-powered sensors and actuators [291].

When incorporated into transient BioMEMSs, the devices are specifically designed to perform their function and then safely degrade within the body. They hold significant promise for applications such as temporary implants, wound healing monitors, and biodegradable diagnostic tools [292]. Collectively, these innovations support the advancement of more sustainable, biocompatible, and intelligent biomedical technologies [293].

Glycine and other biomolecular crystals are gaining attention as valuable materials in the development of piezoelectric biomolecular systems and transient BioMEMSs [294]. As the simplest amino acid, glycine can form crystals with a non-centrosymmetric structure, enabling them to exhibit notable piezoelectric behavior. These crystals stand out not only for their ability to convert mechanical energy into electrical signals but also for being inherently biocompatible and biodegradable [295,296]. Such qualities make them especially well suited for self-powered biomedical devices.

When incorporated into transient BioMEMSs, the tiny, implantable systems are designed to perform a specific function and then safely degrade in the body [297,298]. Glycine-based piezoelectric materials open up exciting possibilities for applications like temporary sensors, diagnostic implants, and bioresorbable therapeutic devices [299]. Their natural origin, adaptability, and environmentally friendly profile position them as key building blocks in the advancement of smart, sustainable biomedical technologies.

Transient BioMEMS devices represent a significant advancement in medical technology, offering practical and patient-friendly solutions for a range of healthcare applications [300]. These devices are thoughtfully designed to perform their intended function and then naturally dissolve within the body, eliminating the need for surgical removal and reducing the risk of complications.

Their potential uses are diverse, including real-time monitoring of wound healing, controlled drug delivery, temporary neural activity recording, and post-operative care. They are also being investigated for roles in cardiovascular monitoring, orthopedic support, and sensing internal physiological changes such as pressure and temperature [301]. Other applications in sensors, nanogenerators and face masks are also being explored [302,303]. By uniting precise performance with biocompatibility and biodegradability, transient BioMEMSs contribute to a more personalized, efficient, and sustainable approach to modern medicine.

Zhang et al. [304] discovered high piezoelectricity in a molecular crystal, HOCH_2_(CF_2_)_3_CH_2_OH [2,2,3,3,4,4-hexafluoropentane-1,5-diol (HFPD)], with a large piezoelectric coefficient of 138 picocoulombs per newton. The HFPD films exhibited excellent flexibility and high biocompatibility, which made it a novel template for the development of transient implantable electromechanical devices.

Yang et al. [305] developed a method for making high-quality crystalline thin films of piezoelectric γ-glycine crystals that are grown and refined between layers of polyvinyl alcohol (PVA). This work presented a straightforward and scalable way to create high-performance piezoelectric biomaterials, paving the way for next-generation, temporary implantable devices that can convert mechanical signals into electrical signals inside the body.

Zhang et al. [306] discovered an active self-assembly strategy to tailor piezoelectric biomaterial thin films. The β-glycine films exhibited an enhanced piezoelectric strain coefficient of 11.2 pm V^−1^ and a piezoelectric voltage coefficient of 252  ×  10^−3^ Vm N^−1^. This finding provided a broadly applicable approach to developing high-performance, large-scale piezoelectric bio-organic materials, supporting the advancement of biological and medical microdevices.

Le et al. [307] reported a reusable, self-sustaining, and humidity-resistant air filtration membrane with high particle-removal efficiency based on highly controllable and stable piezoelectric electrospun PLLA nanofibers. The filter showed long-lasting, stable filtration performance and excellent resistance to humidity, as evidenced by only a slight decrease in filtration efficiency after exposure to moisture. Additionally, the PLLA filter was reusable and was easily sterilized using standard methods such as an ultrasonic cleaning bath, an autoclave, or a microwave.

Wang et al. [308] synthesized a fully biodegradable piezoelectric membrane composed of PVA and glycine via the electrospinning process. The face masks made of the PVA–glycine membrane showed a very high filtration efficiency of 97%, which remained stable over at least ten hours of high-concentration continuous filtration. The biodegradability of PVA–glycine masks was also demonstrated, which could degrade completely within a few weeks, compared to commonly used surgical masks requiring over thirty years to be degraded.

## 3. Wearable Devices

### 3.1. Overview of Wearable BioMEMSs

Wearable BioMEMSs are advanced, miniaturized devices that integrate microfabrication technology, sensors, actuators, and microelectronics to monitor and interact with biological systems in real time. These devices are pivotal in modern healthcare due to their ability to collect continuous, precise physiological data, enabling early diagnosis, personalized treatment, and proactive health management [309]. By seamlessly integrating with the human body, wearable BioMEMSs facilitate non-invasive monitoring of critical biomarkers such as glucose levels, heart rate, and blood pressure [310]. This capability supports chronic disease management, enhances preventive care, and reduces the need for frequent clinical visits. Furthermore, their integration with IoT frameworks allows for secure data transmission for analysis and decision-making, revolutionizing patient care by shifting the focus from reactive treatments to proactive health interventions [29].

Wearable BioMEMSs offer several key benefits over conventional medical monitoring systems. One of the most significant advantages is their ability to provide continuous and real-time monitoring of vital signs and health metrics, allowing for early detection of potential health issues and timely interventions [5]. Unlike traditional monitoring methods, which often require periodic hospital visits or manual data collection, wearable devices enable remote monitoring, reducing the need for frequent clinical check-ups and enhancing patient convenience [311]. Additionally, these devices facilitate personalized healthcare by generating vast amounts of data that can be analyzed using AI and ML algorithms to tailor treatment plans to individual needs [312]. This approach not only improves patient outcomes but also helps in managing chronic diseases more effectively by providing continuous feedback and adjustments to treatment strategies. Furthermore, wearable devices can reduce healthcare costs by minimizing hospitalizations and emergency interventions through proactive monitoring and management.

The wearable BioMEMS market is experiencing rapid growth, fueled by advancements in technology and increasing consumer demand for personalized healthcare solutions. With the wearable healthcare devices market projected to reach USD 69.2 billion by 2028, the digital revolution has firmly established wearable technology as the next frontier in healthcare innovation [313]. Leading tech companies like Microsoft, Apple, and Samsung are actively investing in wearable device development, introducing products aimed at enhancing health monitoring and fitness tracking capabilities.

**Figure 1:**
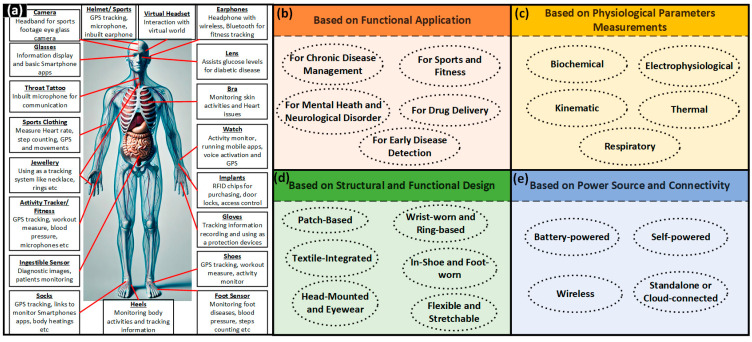
(**a**) Placement and types of wearable devices on the human body, illustrates various locations and their corresponding functionalities. Types of wearable BioMEMSs based on their (**b**) functional applications in healthcare and wellness, (**c**) physiological parameters, (**d**) design, and (**e**) source and connectivity.

Figure 1 provides a comprehensive overview of wearable BioMEMS technology, segmented into five distinct categories, each highlighting a unique aspect of these innovative devices. Figure 1a, focusing on the placement and types of wearable devices on the human body, illustrates various locations and their corresponding functionalities. Head-mounted devices like cameras and virtual headsets offer capabilities such as capturing sports footage, providing augmented reality interactions, and enabling virtual world engagement, while earphones facilitate wireless audio and fitness tracking. Eyewear like glasses displays information and integrates basic smartphone applications, and contact lenses assist with continuous glucose-level monitoring for diabetic patients. A throat tattoo incorporates a microphone for discreet communication.

Sports clothing monitors heart rate, step count, GPS data, and movements during physical activities. Jewelry such as rings and necklaces serves as a tracking system. Activity trackers and fitness bands measure GPS location, workout metrics, blood pressure, and ambient sound levels. Ingestible sensors provide diagnostic imaging and patient monitoring from within the body, while socks link to smartphones to monitor activities, body heat, and GPS location. Other placements include watches for general activity monitoring and implants (like RFID chips) used for purchasing, door locks, and access control. Gloves serve to track information, record data, and provide protection. Shoes and heels can track steps, measure workout metrics, and provide activity monitoring. Finally, foot sensors monitor foot diseases, blood pressure, and step counts.

Figure 1b classifies wearable BioMEMSs based on their functional applications in healthcare and wellness. Wearables are categorized for chronic disease management, targeting conditions like diabetes and cardiovascular diseases, as well as for sports and fitness, tracking hydration, fatigue, and muscle activity. Other categories include mental health and neurological disorder monitoring for stress, sleep, and brain activity; drug delivery for controlled medication administration; and early disease detection to identify conditions like sepsis, infections, or cancer in their early stages.

Figure 1c categorizes the devices by the physiological parameters they measure. Biochemical wearables detect molecular biomarkers such as glucose, lactate, and cortisol. Electrophysiological wearables measure electrical signals from the body, including ECG, EEG, and EMG. Kinematic wearables track motion, posture, and biomechanics with accelerometers and gyroscopes. Thermal wearables monitor body temperature fluctuations, and respiratory wearables assess breathing patterns and lung function.

Figure 1d details the categorization based on design. Patch-based wearables are adhesive skin patches integrating microfluidics, sensors, and microelectronics such as sweat-based glucose monitoring patches, electrochemical tattoo biosensors, and microneedle patches for drug delivery. Textile-integrated wearables include smart textiles with biosensors and microfluidic channels, like e-textile ECG monitoring shirts, sensor-integrated compression sleeves for hemodynamic monitoring, and glucose-monitoring socks for diabetic patients.

Head-mounted and eyewear BioMEMSs include smart contact lenses for intraocular pressure (IOP) monitoring, EEG-integrated headbands for brain activity tracking, and augmented reality (AR) glasses with biosensors for fatigue monitoring. Wrist-worn and ring-based BioMEMSs are compact devices for real-time biosensing, like smartwatches for continuous glucose monitoring (CGM), ring-based pulse oximeters and heart rate trackers, and electrodermal activity (EDA) sensors for stress monitoring. In-shoe and foot-worn BioMEMSs embed sensors in footwear for gait analysis and pressure distribution sensing, such as smart insoles for diabetic foot ulcer prevention, footwear-based motion sensors for fall risk detection, and pressure-sensitive socks for neuropathy management. Lastly, flexible and stretchable BioMEMSs are ultra-thin, stretchable sensors for continuous biophysical and biochemical monitoring, like skin-mounted stretchable hydration sensors, flexible ECG patches with wireless communication, and bioelectronic tattoos for monitoring lactate levels.

Lastly, Figure 1e classifies wearable BioMEMSs based on their power source and connectivity. Battery-powered wearables use standard rechargeable batteries, while self-powered wearables harvest energy through piezoelectric, thermoelectric, or triboelectric means. Wireless wearables use Bluetooth, NFC, or Wi-Fi for data transmission. Devices are also categorized as standalone, functioning independently, or cloud-connected, syncing with cloud platforms for enhanced data management.

### 3.2. Types of Wearable BioMEMSs

To gain a deeper understanding of the diverse range of wearable BioMEMSs, we will categorize them based on their core technology and operational principle. This approach shifts the focus from application-specific functionalities or structural designs to the fundamental mechanisms allowing these wearables to interact with the human body and gather or deliver physiological information or therapeutic agents. This perspective offers valuable insights for engineers, developers, and researchers involved in advancing BioMEMS technology.

#### 3.2.1. Wearable Biosensors

Wearable biosensors have seen significant advancements in detecting biochemical markers like glucose, lactate, and cortisol, enabling real-time health monitoring and disease management through non-invasive methods. These devices employ diverse detection mechanisms, with electrochemical biosensors gaining prominence due to their high sensitivity and specificity. By measuring electrical signals generated during biochemical reactions, these sensors excel in continuous monitoring applications. For example, continuous glucose monitors (CGMs) use electrochemical reactions to track glucose levels in interstitial fluid, offering vital insights for diabetes management [314,315].

**Figure 2:**
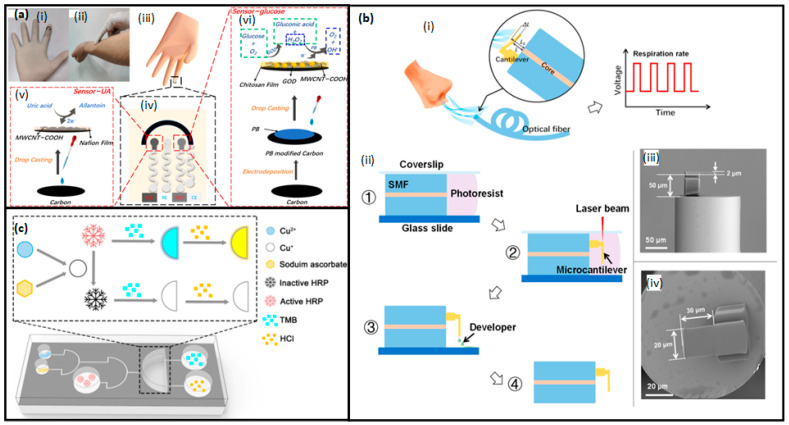
(**a**) Dual-purpose electrochemical wearable sensor for the detection of glucose and uric acid, (i) optical representation of the dual-purpose wearable electrochemical sensor. (ii) Optical representation of the “touch-and-sense” concept. (iii) Diagram of the wearable sensor printed on a rubber glove. (iv) Diagram of the four-electrode sensor. (v,vi) Diagrams illustrating the production of sensor–UA and sensor–glucose, together with their respective sensing mechanisms for uric acid (UA) and glucose. Copyright 2023 MDPI. Reproduced with permission from Ref. [322]. (**b**) (i) Proposed breath sensor schematic. The fiber-tip microcantilever deflects and recovers during inhale and exhale. (ii) TPP technology-based fs laser microfabrication of a fiber-tip microcantilever. (iii,iv) SEM pictures of the device. Copyright 2022 MDPI. Reproduced with permission from Ref. [323]. (**c**) The detection mechanism of the fabricated enzyme method-based microfluidic chip. Copyright 2021 MDPI. Reproduced with permission from Ref. [324].

The field of wearable electrochemical biosensors has seen significant advancements in recent years, particularly with the integration of two-dimensional nanomaterials for health monitoring and clinical analysis, as shown in Figure 2a [316]. The development of multifunctional inks, such as Prussian Blue/graphene ink, has enabled the creation of flexible biosensors and supercapacitors, showcasing the versatility of these technologies [317]. By leveraging nanomaterials and cross-cutting technologies, researchers have designed smart and sensitive electrochemical biosensors capable of detecting chemical warfare agents, emphasizing the need for rapid and customizable detection methods [318]. Wearable electrochemical biosensors have also been explored for measuring biomarkers with complex blood-to-sweat partition, such as proteins and hormones, highlighting the importance of close contact between the biomarker, biorecognition element, and electrode surface for accurate detection [319]. Innovations like hollow microneedle sensing patches have been developed for transdermal electrochemical monitoring of glucose, demonstrating the potential for non-invasive biomarker monitoring [320]. Recent innovations have led to the development of ultra-small wearable biosensor systems capable of continuous sweat analysis, enabling real-time monitoring of biomarkers like glucose, lactate, sodium, and potassium [321].

Hydrogel-based wearable electrochemical biosensors have also gained attention because of their mechanical properties and electrochemical behavior, with both conductive and non-conductive hydrogels being explored [325]. These advancements have paved the way for personalized, real-time, and preventive healthcare monitoring, focusing on detecting a wide range of analytes crucial for disease diagnosis and management [326]. Furthermore, universal flexible wearable biosensors have been developed for non-invasive health monitoring, offering accurate and sensitive detection of glucose, uric acid, and lactate in human sweat samples [327]. Additionally, the review paper by Yoon et al. 2025 has provided deep insight into the potential of nanotechnology-based wearable electrochemical biosensors for real-time disease diagnosis [328].

Skin-attachable sensors have recently emerged as a transformative class of wearable BioMEMSs, designed to achieve seamless, conformal integration with the skin for real-time, long-term physiological monitoring [329]. Unlike conventional rigid wearables, these ultrathin and stretchable devices utilize biocompatible materials such as conductive polymers, hydrogels, and nanofibers to minimize motion artifacts and improve user comfort. Chen et al. [330] has summarized several such advances, including a PI/CNT composite aerogel strain sensor with ultrahigh sensitivity (11.28 kPa^−1^) and a wide sensing range for monitoring complex human motion, such as walking and finger flexion.

For cardiovascular health, Wang et al. [331] developed flexible polyvinyl alcohol/cellulose/PEDOT:PSS electrodes capable of capturing high-fidelity ECG signals, while Zhang et al. [332] demonstrated a miniaturized photodetector based on amorphous SiGe junctions for photoplethysmographic heart rate monitoring. Additionally, advanced chemical sensors were highlighted for their ability to non-invasively monitor glucose [333] and cortisol via sweat [334,335], using enzyme-free electrochemical platforms integrated onto flexible substrates.

These skin-attachable platforms are rapidly advancing toward multifunctional systems, combining multiple sensing modalities (e.g., motion [336], temperature [337], bioelectrical [338], ion selective [339,340], and chemical [341]) to deliver continuous health data with clinical relevance. Their growing role in personalized healthcare, rehabilitation, and pandemic-responsive diagnostics underscores their potential as the next generation of unobtrusive, body-integrated medical devices.

Similarly, optical wearable biosensors have made significant advancements in recent years, offering high sensitivity, multiplexed detection, and real-time analysis of biomarkers in biological fluids like sweat, saliva, and interstitial fluid. These biosensors utilize a range of optical mechanisms, including fluorescence, absorbance, reflectance, and Raman scattering, to detect key physiological and biochemical parameters. One of the most promising approaches involves surface-enhanced Raman spectroscopy (SERS)-based wearable plasmonic sensors, which utilize plasmonic nanostructures to amplify Raman signals, enabling single-molecule detection with high specificity [342].

Recent advancements in flexible plasmonic metasurfaces, nanoparticle-enhanced Raman substrates, and stretchable SERS sensors have enabled their integration into wearable formats, including skin patches and textiles. This integration supports applications like continuous health monitoring, chronic disease management, and early disease detection [343,344].

Wearable colorimetric biosensors offer an alternative optical approach by utilizing chromogenic reagents that undergo visible color changes upon interaction with target analytes [345]. These sensors enable non-invasive, real-time monitoring of multiple biomarkers, often utilizing smartphone-based imaging for quantification. Recent developments in microfluidic-based colorimetric sweat sensors have enabled the detection of glucose, lactate, pH, sodium, potassium, chloride, and hydration levels. This is achieved through enzymatic reactions in which substrate interactions produce measurable color shifts [346,347].

Advances in electrochromism (for electron transfer reactions) [348], ionochromism (for ion detection) [349], and halochromism (for pH sensing) [350] have further improved selectivity, ensuring reliable signal generation for specific analytes [351]. The integration of microfluidic paper-based sensors has further enhanced sweat collection efficiency, optimizing fluid flow and reaction sequencing for improved accuracy and usability in wearable applications [345].

Fluorescence-based wearable biosensors have enhanced detection sensitivity by incorporating fluorophores that emit light when excited [352]. These biosensors utilize chemical probes that change fluorescence intensity in response to analyte interactions, enabling real-time monitoring of biomarkers such as chloride, sodium, and zinc ions [353]. Recent innovations in smartphone-compatible fluorescence imaging systems have integrated microfluidic reservoirs embedded with fluorogenic probes, using dark-shielded optical modules with LED-based excitation and emission filters for precise biomarker quantification [354]. The combination of fluorescence-based wearable biosensors and smartphone imaging has expanded their applications to include enzymatic assays, where binding events induce shifts in fluorescence signals, further improving sensitivity and specificity in non-invasive health monitoring [355].

Another innovative optical approach involves optical fiber-based wearable biosensors, as shown in Figure 2b, which have gained traction in health and fitness applications by incorporating polymer optical fibers (POFs) embedded in textiles for physiological and biochemical sensing [356]. These sensors operate on optical power variation principles and provide high linearity, repeatability, and multiplexed detection of parameters such as temperature, pressure, and motion.

Wearable graphene photodetectors integrated with semiconducting quantum dots have enabled non-invasive monitoring of vital health signs, including heart rate, arterial oxygen saturation (SpO_2_), and respiratory rate. These flexible, transparent devices operate under ambient light conditions and offer low power consumption, making them suitable for continuous health tracking. Further developments have combined flexible ultraviolet-sensitive photodetectors with near-field communication (NFC) circuits, allowing for wireless data transfer and battery-free operation, paving the way for seamlessly integrated wearable sensing technologies [353].

Microfluidic integration has significantly enhanced the performance and efficiency of optical wearable biosensors [357]. By incorporating thread-based hydrophilic and hydrophobic modifications, these sensors can precisely control fluid flow and reaction sequencing, ensuring simultaneous, high-contrast signal development for improved analyte detection, as demonstrated in Figure 2c [358]. The integration of smartphone-based fluorescence imaging systems with PDMS-based microfluidic platforms has further streamlined real-time data collection and signal quantification, providing portable, non-invasive monitoring solutions [352]. These advancements have enabled wearable biosensors to evolve beyond simple point-of-care testing toward continuous and real-time tracking of biochemical markers [359].

While optical wearable biosensors are recognized for their superior sensitivity and multiplexing capabilities, they are often compared to electrochemical biosensors, which are widely adopted due to their faster response time, low power consumption, and cost-effectiveness [359]. A comparative analysis of chemiluminescence (CL) and amperometric biosensors revealed that CL-based biosensors offer higher detectability but require longer acquisition time, limiting their suitability for real-time monitoring [355]. However, recent advancements in optical biosensing, particularly in plasmonic SERS, fluorescence imaging, and wearable imaging systems, have significantly enhanced their competitiveness in sweat-based diagnostics and multiplexed biomarker detection [342].

Further, the integration of wireless communication protocols such as Bluetooth, NFC, and Wi-Fi with cloud-based health analytics has enhanced the accessibility of optical wearable biosensors, enabling seamless remote diagnostics and personalized health interventions [309,359]. As advancements continue in nanophotonic materials, flexible bioelectronics, and AI-powered image analysis, optical wearable biosensors are becoming essential tools for precision healthcare, personalized medicine, and non-invasive disease monitoring.

The integration of two-dimensional (2D) materials has revolutionized the field of wearable BioMEMS biosensors, leveraging their excellent mechanical flexibility, biocompatibility, and high carrier mobility. Recent innovations have led to the development of innovative devices such as electronic skins (e-skins), contact lens sensors, smart wristbands, and diabetic patches, showcasing the broad applicability of 2D materials in real-time health monitoring [288]. Materials like graphene [360], transition metal dichalcogenides (TMDCs) [361], black phosphorus [362], and MXenes [363] have shown remarkable potential in biosensing applications, enabling flexible, stretchable, and highly sensitive devices for detecting biomarkers, physiological parameters, and environmental conditions. Among the most recent developments are wearable sensors for continuous monitoring of glucose, pH, electrolytes, and stress biomarkers, as well as wireless and self-powered biosensing systems, improving the usability in clinical healthcare settings [5]. Sun et al. have demonstrated that the development of 2D material-based biosensors has successfully bridged the gap between lab-based sensing and real-world applications, enabling miniaturized and high-performance wearable platforms [364].

Further, the integration of AI into wearable biosensors has significantly improved the real-time monitoring and interpretation of physiological signals such as heart rate (HR), heart rate variability (HRV), and electrodermal activity/galvanic skin response (EDA/GSR). Barki et al. [365] demonstrated the application of deep neural networks on data acquired from the Empatica E4 wristband, achieving a classification accuracy of 96% when combined with self-reports and 88% using wearable data alone.

This device collected HR from PPG, EDA/GSR, and skin temperature to monitor autonomic responses. Similarly, Florea et al. [366] utilized the Zephyr BioHarness 3 and Shimmer sensors to track HRV and EDA/GSR, respectively. Their machine learning model achieved 86% overall accuracy in classifying physiological responses induced during the Maastricht Acute Stress Test. Kong et al. [7] employed data from Samsung Gear smartwatches and the Empatica E4 to perform stress classification using HRV, EDA/GSR, and skin conductance (SC) features.

Their study highlighted the effectiveness of random forest models, achieving F1 scores of above 65% despite limitations posed by real-life noise and participant variability. These studies exemplify how AI-powered wearable BioMEMS platforms are evolving from static monitors into context-aware and intelligent health companions capable of processing multimodal biosignals for accurate physiological state assessment.

The integration of AI into wearable biosensors enables enhanced real-time monitoring and interpretation of physiological signals, including heart rate (HR), heart rate variability (HRV), and electrodermal activity/galvanic skin response (EDA/GSR). Supervised machine learning models, such as random forest (RF), support vector machines (SVMs), and decision trees, are frequently employed to analyze data from wearable biosensors due to their interpretability, low computational demands, and effectiveness with structured physiological data. The success of these models depends on high-quality input features and consistent, labeled data [367].

Betti et al. [368] is among the early studies to integrate multi-sensor physiological monitoring with AI, demonstrating the benefit of combining HRV, EDA, and EEG for improved prediction accuracy. The use of PCA enabled the model to focus on the most informative features, reducing overfitting despite the small dataset. Notably, the study highlights the importance of correlating AI predictions with biochemical markers like cortisol, enhancing model validity beyond self-reports.

Anusha et al. [369] employed a localized supervised learning approach with adaptive dataset partitioning to classify stress levels in patients prior to surgery, using data collected from the ADI-VSM wearable device. The primary biosignal used was electrodermal activity (EDA/GSR), and the model aimed to categorize participants into low, moderate, or high levels. Ground truth labels were obtained from both the State–Trait Anxiety Inventory (STAI) and salivary cortisol levels. The model achieved 97.83% accuracy with person-specific factors and 85.06% without personalization.

Barki and Chung’s [365] approach exemplifies how deep learning—particularly CNNs—can be applied to physiological data from wearable sensors for automatic, real-time recognition. It also shows that even compact sensors (like in-ear wearables) can be integrated with AI models to detect states without invasive monitoring or active user input. Aristizabal et al. [370] demonstrate how multimodal biosensor data—when combined with deep learning models and statistical handling of individual variance (GEE)—can yield high-accuracy detection. They go beyond signal classification by correlating AI inferences with objective biomarkers like cortisol, which is rare in wearable AI studies.

To better appreciate the diversity and maturity of wearable BioMEMS technologies, it is helpful to examine their practical performance characteristics. These examples span a range of sensing platforms, including electrochemical, optical, piezoelectric, triboelectric, and microfluidic systems, used for noninvasive health monitoring through sweat, breath, or interstitial fluid. Table 6 summarizes recent representative wearable BioMEMS devices with quantitative performance metrics, including sensitivity, limit of detection (LOD), and response time, offering a comparative snapshot of current advancements in the field.

#### 3.2.2. Wearable Bioelectronic Devices

Wearable bioelectronic devices have revolutionized healthcare by providing real-time monitoring of electrical activity in the body through non-invasive, compact, and highly sensitive systems. These devices, which include electrocardiography (ECG), electroencephalography (EEG), and electromyography (EMG) sensors, utilize electrophysiological sensing to detect and record bioelectrical signals generated by physiological processes [375]. By leveraging novel materials, flexible substrates, and wireless communication technologies, these devices enable continuous, non-invasive health tracking [376,377]. The integration of flexible, non-invasive electrodes and advanced data processing techniques has significantly enhanced their accuracy, usability, and overall impact on healthcare monitoring [378].

**Figure 3:**
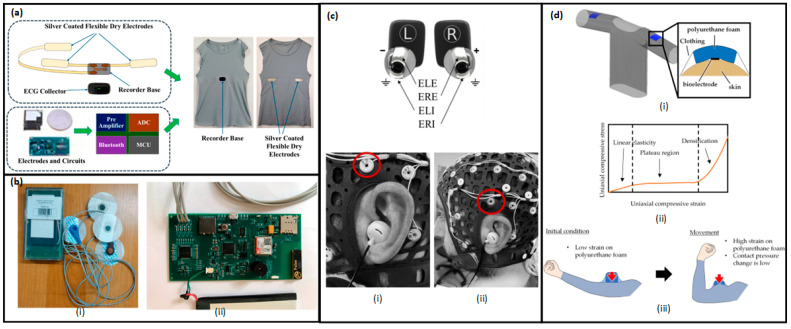
(**a**) Wearable smart T-shirt ECG acquisition system used in this investigation. Wearable smart T-shirts have three silver-coated dry electrodes, a smart textile, a recorder base, and an ECG collector. Display devices like smartphones, PCs, laptops, and tablets communicate ECG signals over Bluetooth. Copyright 2022 MDPI. Reproduced with permission from Ref. [382]. (**b**) A prototype of a wearable ECG device developed at Satbayev University: (i) external view; (ii) board. Copyright 2024 MDPI. Reproduced with permission from Ref. [383]. (**c**) Recording setup for this study. (Left) Naox Technologies in-ear device earpieces with electrodes in ELE, ERE, ELI, and ERI, with two contact points per ear canal. (Right) Left side of dual configuration, with scalp and in-ear devices. (i,ii) Comparisons used standard scalp T7 electrode (red circle). Copyright 2024 MDPI. Reproduced with permission from Ref. [384]. (**d**) (i) Polyurethane foam wearable EMG measurement device construction. (ii) Polyurethane foam plateau. (iii) Using polyurethane foam to stabilize contact pressure. The figure shows the urethane foam and garments’ force direction with red arrows. Copyright 2024 MDPI. Reproduced with permission from Ref. [385].

For ECG monitoring, researchers have explored flexible and stretchable electrodes to enhance signal fidelity while ensuring user comfort, as shown in Figure 3a. Alberto et al. [379] developed an electronic tattoo incorporating liquid metal-based electrodes, which significantly improved interface capacitance and reduced signal noise compared to traditional Ag/AgCl electrodes, as shown in Figure 3b. Similarly, Rachim et al. [380] introduced a graphene-based epidermal patch fabricated via laser-induced transformation of polyimide (PI) into conductive graphene, demonstrating comparable performance to standard ECG electrodes. Additionally, Chung et al. [381] proposed a carbon-black/PDMS-based soft electrode designed specifically for neonatal and pediatric ECG monitoring, which maintained high biocompatibility and conformability while ensuring minimal skin irritation.

Casson et al. [386] has provided an extensive review of wearable EEG devices, compiling a comprehensive assessment of sensor technologies, signal processing techniques, and emerging applications in real-time brain monitoring. Building on this foundation, recent research has focused on enhancing signal acquisition while minimizing motion artifacts. Xu et al. [387] developed an in-ear EEG sensor array that integrates lactate biosensing for seizure differentiation, leveraging a screen-printed electrode system for enhanced stability. Conventional scalp EEG electrodes face challenges due to signal attenuation caused by the skull’s impedance, as demonstrated in Figure 3c. However, wearable EEG devices using flexible hydrogel-based silver electrodes, such as those developed by Carvalho et al. [388], offer a promising solution for long-term monitoring. This new generation of EEG sensors provides a non-invasive alternative to traditional brain monitoring methods, maintaining high signal accuracy while ensuring comfort and wearability.

Both conventional electromyography and surface electromyography (sEMG) [389] have seen significant enhancements in EMG applications through the use of advanced materials and flexible architectures, which are shown in Figure 3d. Researchers have explored the integration of carbon nanotube-based electrodes to improve the sensitivity and conformability of EMG patches [390]. Additionally, innovative designs incorporating serpentine gold mesh electrodes and wireless communication modules have shown promise in real-time muscle activity tracking, with minimal signal distortion [391,392]. The developments in EMG biosensors have significant implications for rehabilitation monitoring, prosthesis control, and the diagnosis of neuromuscular disorders.

One of the most significant advancements in wearable bioelectronics has been the development of flexible, biocompatible electrodes. These innovations have improved signal fidelity, comfort, and long-term usability, addressing the limitations of traditional rigid electrodes, which often cause skin irritation and hinder continuous health monitoring [393]. The emergence of PEDOT:PSS-based bioelectronic materials, as highlighted by Zhang et al. [394], has been particularly noteworthy. These materials offer high conductivity, flexibility, and aqueous processability, making them well suited for wearable ECG, EEG, and EMG devices. Additionally, Alberto et al. [379] and Rachim et al. [380] have introduced liquid metal circuits and graphene-based epidermal patches, which enhance signal quality and stretchability, allowing for seamless integration into bioelectronic systems.

Despite the significant advancements in wearable biosensors, there is still a pressing need for further research into novel electrode materials that provide enhanced biocompatibility, durability, and long-term stability for continuous health monitoring applications [395]. Another groundbreaking development is the integration of AI and ML into wearable biosensors, which has substantially improved biosignal processing, real-time monitoring, and predictive analytics. AI-driven signal processing algorithms have enhanced ECG accuracy, allowing for the early detection of cardiac abnormalities, even during activities that involve a lot of movement [396].

EEG biosensors have harnessed the power of AI to enhance brain–computer interfaces (BCIs) and mental health diagnostics, achieving accuracy rates exceeding 90% in detecting memory responses and emotional states. Similarly, AI-enhanced EMG and surface EMG (sEMG) processing enables precise muscle activity tracking, which is crucial for rehabilitation, prosthetic control, and silent speech recognition. These advancements have positioned wearable bioelectronics as intelligent, adaptive health systems that integrate advanced materials, multimodal biosensing, and AI-powered analytics to deliver real-time, personalized healthcare solutions.

Another transformative advancement has been the integration of AI and ML into wearable bioelectronic systems, revolutionizing biosignal processing, real-time monitoring, and predictive analytics. AI-driven signal processing algorithms have significantly improved ECG analysis, enabling early detection of cardiac abnormalities, including arrhythmias and ischemic events. AI-based motion artifact compensation has further refined ECG accuracy, even during physical activities, enhancing reliability for remote patient monitoring. EEG biosensors have also benefited from AI applications, particularly in brain–computer interfaces (BCIs) and mental health diagnostics.

AI-powered EEG classifiers have demonstrated over 90% accuracy in detecting short-term memory responses and emotional states, highlighting their potential in cognitive health assessment [397]. Similarly, AI-enhanced EMG and surface EMG (sEMG) processing enables more precise muscle activity tracking, supporting rehabilitation monitoring, prosthetic control, and speech recognition applications [398]. Recent research has also explored the integration of wavelet decomposition and ML models for silent speech recognition, allowing real-time gesture-based communication through sEMG patches [399].

#### 3.2.3. Wearable Drug Delivery Devices

**Figure 4:**
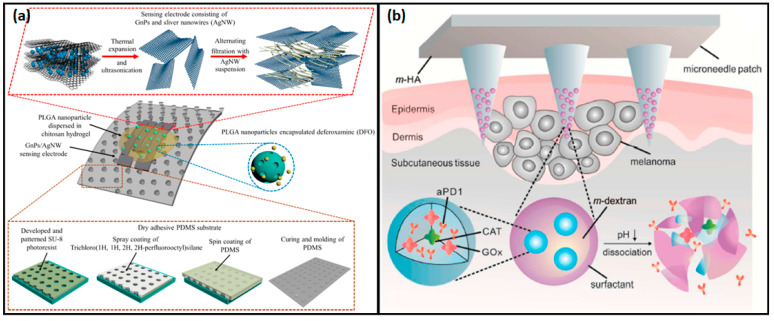
(**a**) Schematic of the multifunctional sensor with flexible strain-sensing and transdermal drug delivery. GnPs, graphene nanoplatelets; AgNWs, silver nanowires; PLGA, polylactic-co-glycolic acid; PDMS. Copyright 2019 MDPI. Reproduced with permission from Ref. [408]. (**b**) Schematic of nanoparticle-loaded microneedle patch with anti-PD-1 (programmed death-1 pathway) delivery. The glucose oxidase/catalase (GOx/CAT) enzyme system encapsulated inside nanoparticles converted blood glucose to gluconic acid, dissociating nanoparticles and releasing aPD1. Copyright 2022 MDPI. Reproduced with permission from Ref. [409].

Wearable drug delivery devices have revolutionized the way medications are administered, offering a non-invasive and controlled approach that enhances patient compliance and therapeutic outcomes. Significant progress has been made in integrating microfluidic and transdermal systems into wearable formats, allowing for precise drug administration across various medical applications [400]. One promising technology is microneedle (MN) systems, which provide a minimally invasive, painless, and efficient method for delivering therapeutic agents through the skin, as shown in Figure 4a,b [401].

Microneedle (MN) patches consist of microstructured arrays that penetrate the stratum corneum without reaching deeper nerve endings, reducing discomfort while facilitating targeted drug administration. Recent advancements in MN applications have shown their effectiveness in delivering insulin for diabetes management, offering a viable alternative to traditional subcutaneous injections [402,403]. The development of biodegradable and dissolvable microneedles has significantly enhanced patient compliance by eliminating the need for patch removal and minimizing medical waste [404,405]. The integration of polymeric and hydrogel-forming microneedles has significantly expanded their potential for controlled and sustained drug release, transforming them into a powerful tool in therapeutic applications [371].

In recent years, iontophoresis-based wearable drug delivery systems have seen significant advancements, particularly with the integration of microneedle arrays and electroporation-assisted technologies. Yang et al. developed a smartphone-powered iontophoresis–microneedle patch that enables controlled transdermal drug delivery with real-time monitoring [406]. Similarly, Liu et al. [407] introduced a mesoporous microneedle–iontophoresis system designed for diabetes management, which demonstrates enhanced drug permeability and precise dosing control. The combination of iontophoresis and dissolving microneedles has been particularly effective in delivering biologics and macromolecules, allowing for minimally invasive administration with improved patient compliance.

Recent studies have highlighted the potential of AI-driven adaptive iontophoretic systems, where ML algorithms optimize drug delivery parameters based on skin impedance and physiological responses. For instance, Amano et al. [410] explored the use of iontophoresis for delivering pilocarpine, showcasing its effectiveness in sweat diagnostics and neurostimulation applications. Meanwhile, Zheng et al. [411] demonstrated the potential of iontophoresis-driven microneedle patches for actively delivering vaccine macromolecules transdermally, expanding the scope of wearable therapeutic technologies. Despite these advancements, challenges such as skin irritation, electrode optimization, and maintaining consistent drug permeation across different skin types remain active areas of research [412]. The integration of AI in monitoring is expected to revolutionize personalized transdermal drug delivery, ensuring precision medicine with enhanced therapeutic efficacy [413]

Smart inhalers represent a major breakthrough in wearable drug delivery systems, specifically tailored to help patients manage respiratory conditions like asthma and chronic obstructive pulmonary disease (COPD). These innovative devices are equipped with sensors that monitor medication usage patterns, offer real-time feedback, and remind patients to stick to their prescribed treatment plans (e.g., Teva Pharmaceuticals’ ProAir^®^ Digihaler™ and ResMed’s Propeller Health) [414]. The data collected by these devices can be shared with healthcare providers, facilitating remote monitoring and allowing for timely medical interventions.

Some smart inhalers have the capability to assess lung function, providing comprehensive insights into disease progression and treatment efficacy. Recent advancements have concentrated on enhancing connectivity, user engagement, and seamless integration into digital health ecosystems, which in turn improve patient outcomes and optimize treatment strategies (e.g., Adherium’s Smartinhaler™ and CoheroHealth’s HeroTracker^®^). However, challenges such as device compatibility, maintenance needs, and patient engagement remain areas that require further innovation and clinical validation [415].

The integration of wearable drug delivery devices with wireless body area networks (WBANs) has significantly enhanced real-time monitoring, improved patient compliance, reduced healthcare costs, and increased accessibility to healthcare services [414]. WBANs facilitate seamless communication between wearable or implantable devices and external systems, optimizing drug delivery management. However, challenges persist, including concerns over data security, patient privacy, and device interoperability [416]. Ongoing research focuses on ensuring controlled drug release, maintaining device adhesion across different skin types, and integrating closed-loop systems that respond to physiological changes [417]. Future advancements aim to develop fully integrated smart patches with sensing and stimuli-responsive therapeutic modules, paving the way for personalized medicine [176,418].

#### 3.2.4. Wearable Motion and Mechanical Sensors

Wearable motion and mechanical sensors have become indispensable tools for monitoring human movement and biomechanics in everyday environments. By utilizing technologies like inertial sensors, strain sensors, and piezoelectric mechanisms, these devices detect motion, pressure, and mechanical deformation, converting these physical parameters into electrical signals that can be analyzed for biomechanical insights [288,419]). The rapid advancement of these technologies has broadened their applications across multiple fields, including rehabilitation, posture correction, and sports performance monitoring.

In the field of rehabilitation, wearable inertial sensors have become a valuable tool for assessing gait and lower limb biomechanics. These sensors, which typically combine accelerometers, gyroscopes, and magnetometers, offer a portable and cost-effective alternative to traditional motion capture systems. They allow for continuous monitoring of patients in both clinical and home settings [420]. Studies have confirmed the reliability and accuracy of these sensors in capturing biomechanical parameters during walking, facilitating personalized rehabilitation programs and real-time feedback mechanisms [421]. Additionally, soft exoskeletons equipped with proprioceptive sensors and soft actuators have been developed to enhance mobility and reduce muscle fatigue during prolonged walking sessions, demonstrating improved user adaptability and comfort [422].

Advances in wearable sensor technology have also benefited posture correction. For example, Cerqueira et al. [423] have explored the use of sensor-integrated wearables to monitor spinal alignment and detect improper posture during dynamic activities. Devices with calibrated sensor arrays provide real-time feedback through haptic and visual cues, enabling users to maintain optimal posture and reduce the risk of musculoskeletal injuries [424]. These developments highlight the potential of wearable technologies in real-time postural monitoring and corrective interventions [425].

In sports performance, wearable motion sensors have been used to analyze athletes’ biomechanics, providing insights that were previously limited to laboratory settings [426]. Studies have investigated the feasibility of using wearable sensor vests combined with artificial neural network modeling to estimate ground reaction forces and kinematic parameters during various exercises [427,428]. This approach allows for the collection of biomechanical data in natural training environments, supporting the optimization of athletic performance through data-driven interventions [429]. Furthermore, piezoelectric-based sensors have been integrated into wearable systems to enhance sensing capabilities [430]. Ju et al. [431] have examined the efficacy of piezoelectric transducers in detecting mechanical strain and motion, highlighting their applications in structural health monitoring, energy harvesting, and biomechanical sensing.

Despite these advancements, challenges such as data accuracy, motion artifacts, and user comfort remain [432]. Future research focuses on developing more robust algorithms for biomechanical data processing, improving sensor materials for enhanced skin contact, and integrating multimodal sensing technologies for comprehensive health monitoring solutions [433]. Moreover, the integration of wearable sensors with ML techniques has been explored as a means to advance real-time motion analysis and personalized feedback systems, bridging the gap between laboratory research and practical applications [434]. These developments underscore the potential of wearable sensor technologies in revolutionizing human motion analysis across clinical, sports, and industrial applications.

## 4. Implantable Devices

Implantable BioMEMS devices represent a significant leap in medical technology, enabling continuous health monitoring, targeted drug delivery, and precise therapeutic interventions [8]. Unlike wearable devices, which primarily provide external physiological measurements, implantable devices function within the body, offering real-time data acquisition and controlled interventions with minimal patient involvement. These micro-scale systems integrate sensors, actuators, microfluidics, and wireless communication, making them indispensable for managing chronic conditions, neurostimulation, and personalized medicine [3]. Current advancements in biocompatible materials, smart bioelectronics, and wireless power transfer have enabled more efficient, durable, and autonomous implantable solutions [208]. The sections below discussed three critical applications of implantable BioMEMS: (1) continuous health monitoring, (2) targeted drug delivery, and (3) neurostimulation, highlighting their transformative potential in modern healthcare.

### 4.1. Continuous Health Monitoring and Diagnostics

Implantable BioMEMS devices have revolutionized continuous health monitoring and disease diagnostics by enabling real-time, autonomous physiological assessments with minimal patient intervention [8]. Such improvements are important in chronic disease management, personalized medicine, and neuroelectronic applications. The combination of biocompatible materials, wireless energy transmission, and biosensing technologies has driven advancements in next-generation implantable health monitoring devices, which lead to increased precision, patient comfort, and long-term reliability [435]. The current state of the art in continuous health monitoring and diagnostics is summarized in Table 7, which highlights various devices and their applications.

Li et al. [436] provides a comprehensive analysis of biosensors for patient-friendly diagnostics and implantable devices tailored for minimally invasive treatment. Their study explores key considerations in materials selection, device structures, and wireless communication strategies, paving the way for cost-effective, infection-minimized, and personalized healthcare solutions. Similarly, Wu et al. [437] discuss the role of electrochemical biosensors in implantable systems, emphasizing their potential for point-of-care diagnostics, such as glucose monitoring and rapid disease detection, through highly sensitive analyte recognition and real-time readouts.

The rise of neuroelectronic interfaces has further expanded the applications of implantable monitoring. Dimov et al. [438] highlights the increasing interest in electronically interfacing with the nervous system for health diagnostics and therapy. Their work discusses the integration of semiconducting polymers in implantable microelectrodes and wearable brain-monitoring caps, which improve electrical and mechanical properties at the tissue–device interface, enhancing biocompatibility. Complementing this, Kim et al. [439] summarizes the latest advancements in batteryless, wireless implantable biomedical devices designed for continuous physiological signal monitoring. Their study underscores the importance of overcoming biological constraints and energy sourcing challenges to achieve optimal device performance and seamless human–tissue integration.

Cardiovascular monitoring has also benefited significantly from implantable BioMEMS advancements. Herbert et al. [256] introduce a fully implantable, battery-free soft electronic stent system designed for real-time restenosis monitoring. Their system utilizes nanomembrane strain sensors and inductive stents, eliminating the need for additional surgeries and enabling wireless, passive monitoring of arterial health. Additionally, Sang et al. [440] proposed a fluorescence-based biodegradable microneedle sensor array for continuous glucose monitoring (CGM), providing a minimally invasive, pain-free, and bioresorbable alternative to conventional glucose monitoring methods. This innovative system enhances long-term diabetes management while eliminating the need for device removal.

Long-term monitoring of vital biomarkers—such as glucose, oxygen levels, pH, and intracranial pressure—has been made feasible by advancements in implantable biosensor technology [8]. Among the most commercially successful products are continuous glucose monitoring (CGM) systems like Abbott’s Freestyle Libre and Senseonic’s Eversense, which offer real-time glucose monitoring that enhance diabetes control [441]. These devices have significantly improved patient outcomes by allowing immediate intervention and tailored treatment strategies. Ullah et al. [442] developed an in-vitro skin membrane-based glucose biosensor and demonstrated that even a thin 10 µm stratum corneum layer significantly delays sensor response. Their results showed that 90% of the steady-state response was achieved in ~32 min with intact skin, compared to just 5 min when the stratum corneum was removed. This study underscores the importance of considering tissue diffusion kinetics in the design and interpretation of implantable glucose biosensors. Similarly, the CardioMEMS HF System, an implantable wireless pressure sensor, has revolutionized heart failure management by allowing for early detection of hemodynamic changes, thereby reducing hospitalizations and enhancing patient quality of life [443].

Beyond metabolic and cardiovascular monitoring, neural implants have expanded the scope of real-time diagnostics and rehabilitation. Brain–computer interfaces (BCIs) like Synchron’s Stentrode have pioneered the ability to record brain activity and translate it into actionable digital commands, assisting individuals with paralysis in regaining communication and control over external devices [444]. Additionally, implantable electroencephalography (EEG) sensors are advancing seizure prediction and real-time epilepsy management, opening new possibilities for personalized neurological disorder treatments [445]. Together, these developments highlight the transformative impact of implantable technologies in healthcare, underscoring their potential to revolutionize disease management and patient care through continuous, precise, and autonomous monitoring solutions.

Implantable bioelectronics also require sustainable and efficient energy storage solutions. Yu et al. [446] reviews state-of-the-art implantable energy storage devices, emphasizing biocompatible, miniaturized, and biodegradable batteries and supercapacitors tailored for long-term implantable applications. Their study highlights the necessity of safe, stable, and adaptive power solutions to match the dynamic nature of biological tissues. The exploration of biodegradable sensors further aligns with the shift towards sustainable implantable technologies. Janićijević et al. [447] critically assesses the design and development of bioresorbable sensors that function within a programmed period before safely degrading in the body or environment, reducing electronic waste and eliminating the need for surgical removal.

Yang et al. [448] contributes to the advancement of self-healing and adaptive implantable electronics, presenting transformative, thermal-switching, and reconfigurable soft electronics with exceptional stretchability and conductivity. Their study demonstrates the potential for developing highly resilient implantable devices that conform seamlessly to biological tissues, thereby enhancing durability and patient comfort. Overall, recent developments in implantable BioMEMSs for continuous health monitoring underscore a paradigm shift towards minimally invasive, autonomous, and sustainable healthcare technologies [8]. The integration of wireless communication, self-sustaining energy storage, and biodegradable materials will further enhance the functionality and longevity of next-generation implantable monitoring systems, significantly improving patient care and disease management [449].

### 4.2. Implantable Drug Delivery Systems

**Figure 5:**
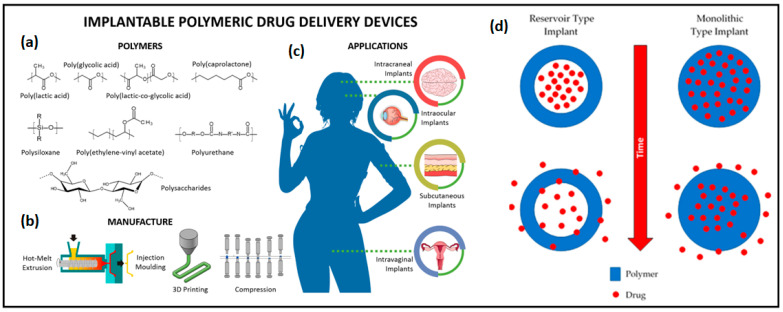
(**a**) An illustration of the chemical structures of various polymers. (**b**) Illustration of various manufacturing processes. (**c**) Illustration of various targets of drug delivery. (**d**) An illustration of reservoir- and monolithic-type implants. Copyright 2018 MDPI. Reproduced with permission from Ref. [451].

Implantable drug delivery systems (IDDSs) have revolutionized drug administration by enabling targeted, sustained, and controlled release of therapeutic agents. Unlike traditional oral and injectable drug delivery, which often results in fluctuating drug concentrations, IDDSs ensure steady-state drug release, improving therapeutic outcomes and patient adherence, particularly for chronic disease management [450]. These systems help circumvent issues such as first-pass metabolism, systemic toxicity, and poor patient compliance, making them an ideal solution for long-term treatment. Given the vastness of this domain, implantable drug delivery could warrant a standalone review paper that comprehensively covers the various technologies, mechanisms, and clinical implementations, as shown in Figure 5a–d. However, in this subsection, we have aimed to provide a precise yet holistic overview, covering fundamental research areas and key base references to establish a foundational understanding. Additionally, we have included Table 8, which summarizes the most recent and advanced implantable drug delivery technologies, offering a snapshot of cutting-edge developments in the field. This table serves as a quick reference for both commercially available and emerging IDDS solutions, highlighting their mechanisms, applications, and technological advancements.

The concept of implantable drug delivery was first introduced by Blackshear (1979) [452], who explored subcutaneous drug-releasing implants and infusion pumps designed to provide continuous drug release. The evolution of these systems was later chronicled by Greatbatch et al. [453], who highlighted the role of industrial advancements in developing implantable biomedical devices, including drug delivery systems and defibrillators. Early implantable devices faced challenges, including drug diffusion limitations, variability in absorption due to surrounding tissues, and poor control over drug release kinetics. Over the decades, significant progress has been made in optimizing these systems for clinical use.

Advancements in biocompatible and functional materials have been pivotal in improving IDDSs. Losic et al. [454] investigated nanoporous materials, emphasizing their advantages in precise drug release and enhanced biocompatibility. Similarly, Ehrick et al. [455] introduced glucose-responsive hydrogel networks, which act as smart gating systems for controlled insulin delivery in diabetes management. Talebian et al. [456] focused on biopolymer-based DDSs for cancer treatment, demonstrating localized, biodegradable drug delivery systems capable of spatiotemporal control over chemotherapy agents. These materials allow for better drug release control, reduced immune response, and longer implant longevity.

**Table 8:** Overview of recent and advanced implantable drug delivery systems (IDDSs).

Device	Function	Technology	Clinical Application	Manufacturer/Ref.
Medtronic Synchromed II	Controlled drug infusion	Programmable pump	Chronic pain, spasticity	Medtronic
MicroCHIPS Biotech Implant	Wireless drug delivery	MEMS-based microchip	Osteoporosis, contraceptive implants	MicroCHIPS Biotech
Bioresorbable drug-eluting implants	Localized drug release	Biodegradable polymers	Cancer, infections	[457]
Nanoporous DDS	Controlled chemotherapy	Nanoporous silicon carriers	Cancer treatment	[458]
Reservoir-type subcutaneous implant	Sustained risperidone release	Biodegradable poly(caprolactone) membrane	Schizophrenia treatment	[459]
Piroxicam microcapsule-embedded scaffold implant	Sustained NSAID release	PLA/PLGA-based gelatin microcapsules in a scaffold	Arthritis pain management	[460]
Artemisinin-loaded PLGA/MSNs Composite nanofibers	Targeted drug delivery	Mesoporous silica nanoparticles (MSNs) and electrospun PLGA nanofibers	Breast cancer treatment	[461]
Imidazolium methacrylate–resorcinol dimethacrylate hydrogel implant	Sustained corticosteroid release	3D-printed photopolymerizable hydrogel with dexamethasone	Inflammatory disease management	[462]
Poly(trimethylene carbonate-co-P-dioxanone) implant	Controlled drug release	Biodegradable PTD copolymer with tunable degradation rate	Long-acting drug delivery	[463]
3D-printed EVA28-based subcutaneous implant	Personalized drug delivery	Fused filament fabrication (FFF) of EVA28 with progesterone	Hormonal therapy (progesterone)	[464]
Nanochannel delivery system (nDS) implant	Wireless, controlled drug release	Silicon nanofluidic membrane with BLE control	Chronic disease management	[465]
3D-printed hydrogel implant for tenofovir	Prolonged antiviral drug release	Bovine serum albumin hydrogel with methylcellulose reinforcement, semi-solid extrusion 3D printing	Hepatitis B and HIV treatment	[466]
Supramolecular hydrogel for glioblastoma therapy	Localized chemotherapy	Peptide-functionalized hyaluronic acid hydrogel with cucurbit [8] uril host–guest interactions	Post-surgical glioblastoma treatment	[467]
Implantable scaffold for pancreatic cancer Immunotherapy	Localized immunotherapy	Biodegradable polymer scaffold for sustained drug release	Pancreatic cancer treatment	[468]
pH and NIR dual-responsive TiO₂ Nanotube implant	Stimuli-responsive drug release	TiO_2_ nanotube arrays modified with polydopamine and Fe^3+^	Osteoporosis treatment	[469]
3D-printed biodegradable poly(ether ester) implant	Personalized drug release	High-resolution MEAM using poly(ether ester) multiblock copolymers	Chronic disease management	[470]
γ-cyclodextrin Hydrogel for josamycin release	Sustained antibiotic release	Crosslinked γ-cyclodextrin hydrogel for prolonged drug delivery	Post-surgical glaucoma treatment	[471]
Fluconazole-loaded chitosan nanoparticle composite film	Localized antifungal therapy	Ionic gelation-based chitosan nanoparticles in gelatin–chitosan composite film	Prosthetic joint infection (PJI)	[472]
Naringenin-loaded PHBV/PLGA implantable rods	Neuroprotective drug release	Poly(3-hydroxybutyrate-co-3-hydroxyvalerate)/PLGA blend via melt- and wet-spinning	Retinal degenerative diseases	[473]

Recent advancements in microfabrication and microelectromechanical systems (MEMSs) have enabled the development of highly sophisticated IDDSs. Tng et al. [474] discussed the challenges of MEMS-based implantable drug delivery, emphasizing their role in precision medicine. Meng et al. [475] further explored nano-fabrication and microfluidics, highlighting programmable drug administration through microscale implants. Uguz et al. 2017 [476] introduced a microfluidic ion pump (µFIP) capable of delivering neurological drugs via electrophoresis, demonstrating targeted cortical drug administration in animal models. These developments have significantly enhanced the precision, efficiency, and adaptability of implantable drug delivery systems.

The rise of 3D printing has enabled customizable, patient-specific drug implants. Arany et al. [477] demonstrated the feasibility of fused deposition modeling (FDM) for manufacturing biocompatible implantable drug delivery systems. Quarterman et al. [478] highlighted the advantages of IDDSs over traditional drug administration, underscoring their ability to overcome first-pass metabolism and maintain therapeutic drug levels. The ability to design and print implants tailored to individual patient needs has been a major step toward personalized medicine.

More recently, Picco et al. [479] and Korelidou et al. [480] have also explored the use of 3D-printed biodegradable implants for sustained drug release. Picco et al. [479] developed olanzapine-loaded implants using robocasting 3D printing, achieving sustained drug release for up to 190 days, which is particularly beneficial for treating schizophrenia. Meanwhile, Korelidou et al. [480] have developed a biodegradable porous PLA/PCL-based membranes for antibiotic-loaded implants, demonstrating localized tetracycline release for up to 25 days to control post-surgical infections. Al-Litani et al. [481] highlighted the potential of 3D-printed implantable drug delivery systems in women’s health, while also emphasizing the regulatory challenges and future clinical translation. This underscores the role of customizable 3D-printed implants in providing precise and effective long-term therapy.

The clinical success and regulatory approval of IDDSs highlights their real-world impact. Fayzullin et al. [482] conducted a comprehensive review of FDA-approved IDDS applications, examining their efficacy, safety, and effects on foreign body response. These systems have been effectively used to treat a wide range of conditions, including chronic pain management, hormone therapy, neurological disorders, and cancer treatment. Magill et al. [450] further categorized IDDSs into systemic versus localized drug delivery, illustrating how implants can enhance site-specific drug accumulation while reducing systemic toxicity. In the field of diabetes management, Zhang et al. [483] examined closed-loop glucose monitoring and insulin delivery systems, integrating wearable glucose sensors with implantable insulin pumps. Their review emphasized noninvasive, real-time glucose monitoring, paving the way for automated diabetes care. Advanced smart implants can detect glucose fluctuations and release insulin accordingly, improving glycemic control and patient adherence.

For ocular diseases, Mostafa et al. [484] focused on long-acting drug delivery systems (LADDSs), particularly implantable ocular inserts, which provide steady-state drug release while bypassing ocular barriers. Despite their efficacy, nonbiodegradable implants remain highly invasive, necessitating biodegradable alternatives for long-term ocular therapy. Implants designed for sustained drug release in the eye have shown promise in treating conditions such as glaucoma, macular degeneration, and diabetic retinopathy.

Additionally, MicroCHIPS Biotech has played a pioneering role in programmable drug release. Their implantable microchip technology, which can be activated wirelessly, enables controlled, long-term drug administration, reducing the need for frequent injections [485]. The company has developed implantable contraceptive devices with a lifespan of ~16 years, supported by funding from the Bill & Melinda Gates Foundation. In collaboration with Teva Pharmaceuticals, MicroCHIPS is also working on implants for chronic disease management, including osteoporosis, diabetes, multiple sclerosis, and pain management. Their technology represents a major breakthrough in autonomous, adjustable, and patient-friendly drug delivery [18].

Bioelectronic medicine is another emerging field that integrates implantable stimulation-based therapies with drug delivery. Vagus nerve stimulation (VNS) and spinal cord stimulation (SCS) have demonstrated neuromodulation-based therapy for conditions like chronic pain, epilepsy, and inflammation. By modulating the nervous system with electrical signals, these systems offer an alternative to pharmaceutical treatments, reducing reliance on opioids and systemic medications.

The future of implantable drug delivery lies in the development of smart, autonomous systems capable of real-time physiological feedback and programmable release mechanisms. Advancements in biodegradable materials, nanotechnology, and wireless drug delivery will continue to refine these systems [486]. However, challenges remain, including foreign body reactions, biocompatibility, regulatory hurdles, and scalability for widespread clinical adoption. Research is ongoing to integrate wireless and energy-harvesting technologies that can power long-term drug implants without battery replacements [487].

### 4.3. Neurostimulation and Bioelectronic Implants

Neurostimulation and bioelectronic implants have emerged as transformative technologies in medical science, enabling precise modulation of neural activity to treat various conditions, including chronic pain, epilepsy, neurodegenerative diseases, and cardiovascular disorders. These implants leverage electrical stimulation and bioelectronic interfaces to interact with the nervous system, offering alternative or adjunctive therapies where pharmaceuticals may be inadequate or have significant side effects. The rapid evolution of miniaturized electronics, wireless power transmission, and biocompatible materials has significantly enhanced the capabilities and longevity of neurostimulation devices.

To provide a structured overview of the rapidly evolving landscape of neurostimulation and bioelectronic implants, Table 9 categorizes various devices based on their commercial availability, regulatory approval status, and ongoing research. This table serves as a quick reference, detailing their function, underlying technology, and clinical applications. Given the diversity of neurostimulation systems—ranging from deep brain stimulators to neural recording, spinal cord stimulation to seizure control, and peripheral nerve stimulation to neuromodulation—such a summary is essential for understanding both established and emerging innovations in the field.

The miniaturization of implantable neurostimulation devices presents a significant challenge in terms of power supply. Dinis et al. [491] highlighted how advancements in microfabrication, sensor miniaturization, and wireless communication have enabled the development of compact implantable bioelectronic devices. However, since battery technology has not scaled down at the same rate, alternative power sources are needed. Their review examined various emerging approaches, including energy harvesting and wireless power transfer (WPT), to mitigate battery dependence and extend device longevity.

In addition to wireless energy transfer technologies, biofuel cells (BFCs) have emerged as a compelling strategy for powering implantable bioelectronic systems [492]. These devices convert biochemical energy from naturally available substrates such as glucose, lactate, or oxygen into electrical energy via enzymatic or microbial redox reactions. Within these systems, conducting polymers (CPs)—notably polypyrrole (Ppy), polyaniline (PANI), and PEDOT—play multifunctional roles, including acting as biocompatible matrices for enzyme immobilization, facilitating electron transfer, and enhancing the electrochemical performance of bioelectrodes [493].

Ramanavicius et al. [494] have explored the role of CPs in enabling stable and efficient charge transport within both enzymatic and microbial BFC architectures, highlighting their ability to support redox reactions and interface with biological components under physiological conditions. These polymers can be further engineered into nanostructured or hydrogel formats to improve surface area, mass transport, and mechanical compatibility with soft tissues.

Their compatibility with standard microfabrication methods also makes them suitable for integration into miniaturized BioMEMS platforms. Although the long-term power stability and current output of such systems remain areas of ongoing research, the incorporation of CP-based BFCs offers a promising direction toward reducing dependence on conventional batteries for next-generation implantable devices.

Singer et al. [495] further explored six wireless power transfer (WPT) techniques tailored for bioelectronic implants: inductive coupling, radio frequency (RF) transfer, mid-field transfer, ultrasound, magnetoelectric power, and light-based power delivery. Each method involves tradeoffs between power efficiency, miniaturization, depth of implantation, and alignment tolerance. The study emphasized the importance of hybrid power solutions that combine multiple techniques to optimize energy transfer for different biological targets. The integration of WPT into next-generation neurostimulation devices is essential to enable fully implantable, battery-free, and autonomous bioelectronic systems.

Bidirectional neuromodulation systems have gained attention for their ability to both record and stimulate neural activity, allowing real-time closed-loop interventions. Wright et al. [496] developed a fully implantable wireless neuromodulation system for preclinical studies in mice. Their device, measuring only 2.2 cm^3^ and weighing 2.8 gm, allows simultaneous physiological signal readout and complete control over stimulation parameters. A key advancement in their study was the demonstration of vagus nerve stimulation (VNS) to induce acute bradycardia, with a functional lifespan exceeding three weeks. By designing the implant with commercially available electronic components and 3D-printed packaging, the study aimed to accelerate the translation of neuromodulation systems into clinical applications.

Magisetty et al. [497] examined the broader landscape of electroceuticals, a new class of bioelectronic medicine that encompasses pacemakers, neural stimulators, artificial retinae, and VNS-based implants. They discussed the micro-/nanoscale features and material innovations that have transformed bioelectronic devices from a conceptual stage to clinically impactful therapies. Their study also emphasized the importance of wireless power supply and ultra-low power consumption, which enable multifunctional smart implants capable of real-time monitoring and therapeutic intervention.

Neurostimulators have emerged as groundbreaking implantable BioMEMSs, modulating neural activity to treat various neurological disorders. Deep brain stimulation (DBS) is one of the most well-known applications, where electrodes implanted in specific regions of the brain deliver controlled electrical impulses to alleviate motor symptoms in patients with Parkinson’s disease. Similarly, cochlear implants restore hearing by directly stimulating the auditory nerve, enabling individuals with severe hearing loss to perceive sound. The miniaturization of implantable neuromodulation devices has also enabled the development of peripheral nerve stimulation (PNS), which is currently being explored for conditions such as migraine management, sleep disorders, and cardiac arrhythmias. Future advancements in PNS and neurostimulation technologies focus on wireless and battery-free neuromodulation, leveraging energy-harvesting techniques to extend device longevity and improve patient outcomes.

One of the major challenges in neurostimulation implants is maintaining stable interfaces with biological tissue over long periods. Pak et al. [498] investigated thin-film encapsulation strategies for liquid crystal polymer (LCP)-based bioelectronic implants, comparing multiple coating materials. Their findings demonstrated that while water vapor transmission rates (WVTRs) are often used as a metric for encapsulation efficiency, the adhesion of the coating to the implant substrate plays a more crucial role in maintaining device stability and extending implant lifespan. Their study provided a pathway for improving the biocompatibility and longevity of neurostimulation implants, ensuring they remain functional for extended periods in vivo.

Traditional neurostimulation implants often require invasive surgeries to place electrodes in direct contact with target nerves. Chen et al. [499] proposed an alternative approach with the development of a wireless, battery-free, millimetric magnetoelectric implant for endovascular nerve stimulation. The implant, measuring just 1 mm × 0.8 mm, leverages magnetoelectric materials to wirelessly receive power and stimulation signals.

Their study demonstrated successful wireless stimulation of the sciatic and femoral nerves in both rat and pig models, proving that minimally invasive bioelectronic implants could one day replace traditional surgical implants for neuromodulation therapies.

Long-term implantation of bioelectronic devices is often limited by foreign body reactions that impair recording and stimulation efficacy over time. Boys et al. [500] addressed this issue by developing 3D bioelectronics with a remodelable matrix, which promotes tissue integration while minimizing immune response. Their hybrid implant system, combining microelectrode arrays with bioresorbable gels, demonstrated stable electromyographic recordings in musculature with minimal inflammatory response. This research highlights how biodegradable and bioresorbable coatings could significantly improve the long-term functionality of neural implants.

Peripheral nerve stimulation (PNS) has shown promise in treating neurological disorders and motor disabilities, particularly in amputees. Valle et al. [501] conducted a comprehensive study on the long-term stability of intraneural implants, combining patient data, histological analysis, and computational modeling. Their findings revealed that different electrode configurations and implant positions significantly influence the stability of perceptual thresholds and nerve responses over time. They suggest that future intraneural implants should incorporate adaptive AI-based stimulation algorithms and optimized electrode coatings to address changes in neural sensation over time.

Beyond electrical stimulation, integrating microfluidics with bioelectronic devices opens up new avenues for personalized medicine. Luo et al. [502] reviewed recent advancements in implantable microfluidics, highlighting their potential for targeted drug delivery, biomarker sensing, and regenerative medicine. Their study emphasized the need for further miniaturization and integration of microfluidic components into neurostimulation devices, enabling more sophisticated and adaptive therapies.

AI is increasingly being embedded into implantable BioMEMSs to enable real-time diagnostics, predictive analytics, and adaptive therapy. In cardiac applications, AI-enhanced implantable cardioverter defibrillators (ICDs) and cardiac resynchronization therapy (CRT) systems utilize electrogram data to predict arrhythmic events and personalize treatment strategies.

Algorithms applied to implantable loop recorders have significantly reduced false-positive atrial fibrillation alerts, improving diagnostic precision. Furthermore, models like CHAI integrate clinical, imaging, and electrogram features to provide patient-specific risk assessments for sudden cardiac events [503]. These developments exemplify the shift of implantables from passive monitoring tools to active, AI-driven therapeutic platforms capable of supporting autonomous, personalized care.

The convergence of wireless power transfer, flexible bioelectronics, and adaptive neuromodulation systems is driving the future of neurostimulation implants [10]. As research progresses, the integration of biocompatible coatings, AI-driven closed-loop control, and microfluidic drug delivery will enhance the efficacy, longevity, and patient adaptability of neurostimulation implants [504,505]. Future devices will not only restore lost neurological functions but will also act as intelligent bioelectronic interfaces capable of real-time physiological feedback and intervention [506].

Advances in electrode coatings, biomaterial interfaces, and non-invasive neurostimulation will further optimize implantable neurostimulation technologies, making them more accessible, efficient, and patient-friendly. By leveraging breakthroughs in bioelectronic medicine, soft robotics, and AI-driven biointerfaces, the field of neurostimulation implants is poised to revolutionize neurological disorder treatment, rehabilitation, and precision medicine in the coming years.

## 5. Transforming Healthcare

Wearable devices have reached a milestone in improving health via the monitoring and management of chronic diseases. They provide real-time monitoring of the health of patients and therefore suggest required interventions in a timely fashion [507]. Below are some major fields where wearable health has shown its impact.

### 5.1. Chronic Disease Management

Chronic diseases, also known as noncommunicable diseases (NCDs), are categorized as a long-term condition that needs ongoing medical attention. Some examples of such diseases are heart related conditions (like attacks, strokes), cancers, chronic obstructive pulmonary disease, asthma, and diabetes [508]. According to the World Health Organization (WHO), around 19 million fatalities in 2021 were due to heart related diseases alone, making them a major contributor to NCD-related deaths. The other main contributors are cancer, chronic respiratory diseases, and diabetes, which account for 10 million, 4 million, and more than 2 million deaths, respectively.

Traditionally, patients used to go for periodic checkups and reactive interventions. However, such practices may be inefficient sometimes. In contrast to this, integration of the latest wearable devices in the modern healthcare system seamlessly observes vital signs and health metrics [509]. Such capability empowers both patients and healthcare providers to make timely decisions and enable them to take proactive steps to cease progression and manage symptoms [510,511].

In the field of cardiology, wearable devices are commonly used for monitoring of hypertension, for the detection of arrhythmias [512,513], and to aid in cardiac rehabilitation [514]. Moreover, in case of respiratory health, those devices are frequently used for asthma management [515] and for monitoring of critical parameters. Their neurological applications include seizure detection [516] and management of Parkinson’s disease [517]. Additionally, for endocrinology, wearable devices are used for thyroid dysfunction monitoring [518], fertility tracking [519], and diabetes management [520]. They also play a role in postsurgical recovery in orthopedics and early complication detection in oncology [521]. Additionally, mental health is also taken care of with these devices, such as through anxiety detection and stress reduction through biofeedback [522]. Other examples for wearable technologies include emerging non-invasive monitoring of chronic kidney disease (CKD) [523] and halting kidney disease at the end stage [524]. Despite these advantages, challenges such as data accuracy and privacy concerns persist. However, ongoing innovation and collaboration can maximize the benefits of wearable technologies in healthcare.

### 5.2. Role of Wearable Technologies in Cardiology

Wearable technologies have emerged as indispensable assets in cardiovascular medicine, particularly in managing health conditions such as hypertension. Generally, cardiology-related wearables include fitness trackers, digital blood pressure cuffs, weight scales, and arrhythmia monitoring systems. These devices offer continuous monitoring and personalized insights outside traditional clinical settings.

#### 5.2.1. Monitoring Hypertension

Wearable devices for blood pressure (BP) monitoring are proven to be a superior technique for monitoring cardiovascular disease risk and demonstrate protracted clinical results compared to the traditional office-based BP measurement technique [525]. Wearable devices enable continuous BP measurements without significant disruption to patients’ daily routines. Such an ability is very helpful in identifying masked hypertension and pathological BP variability [526]. Some of the devices, like smartwatches with photoplethysmography, were found to be accurate when compared with standard devices for BP measurement in adults [527]. Moreover, accommodating more features in BP monitoring will provide more details on BP data in real-life scenarios, which will be helpful for medical practitioners to prepare better management strategies for hypertension.

#### 5.2.2. Detecting and Monitoring Arrhythmias

Wearable devices containing optical sensors and photoplethysmography (PPG)-based software algorithms can be useful in the detection of atrial fibrillation (AF). There are some non-invasive and continuous monitoring devices, like ring-type wearable devices such as the CardioTracker (CART) [528] and patch-type devices such as the AT-Patch [529], as well as continuous electrocardiographic patch monitors, that may enhance clinical outcomes and care of patients. Commercially available wrist watches like the Apple Watch, Livmor Halo+ System, and Garmin FitBit, and smartphone apps such as Pulse-Smart, Kardia Mobile, and ECG Check, have proven very useful in arrhythmia detection [530].

Integration of biosensors in wearable devices such as clothes and smartwatches facilitates real-time data of heart rate, BP, ECG, and other biophysical and biochemical signs. These biosensors have improved the management of cardiovascular diseases on both individual and large public scales by enabling remote patient tracking and supporting personalized medicine [531].

Despite the above-mentioned benefits, there are many hurdles, like device accuracy and clinical validation, regulatory policies, privacy considerations, and the imperative for large-scale trials. These challenges somehow cease the widespread implementation of wearable devices in clinical practice. Such issues suggest that collaborative efforts between stakeholders are important to surmount [532].

### 5.3. Role of Wearable Technologies in Respiratory Health

Wearable devices are widely utilized in respiratory health management by ongoing monitoring of respiratory rate, saturation of oxygen, and other important signs. These devices offer a great approach to managing respiratory conditions through early detection of exacerbations and personalized interventions. Wearable respiratory sensors gather real-time datasets on exhaled gas and its components for ongoing monitoring of human health. Such monitoring provides the breathing status of the subject and guides them in the management of conditions like sleep apnea, asthma, and chronic obstructive pulmonary disease. Wearable devices also measure airflow, temperature, and humidity changes in exhaled and inhaled breath to identify breathing patterns [16].

Various wearable devices cater to respiratory monitoring, including chest straps, smart fabrics, and under-the-nose or mask-integrated sensors. Chest straps like the Resmetrix system monitor breathing patterns, heart rate, temperature, activity, and position. Smart fabrics incorporate capacitive stretch sensors to measure chest movements during breathing [533]. Devices like the A-spiro and the RESP^®^ Biosensor facilitate early detection of deterioration [534]. Hexoskin’s sensorized garment monitors heart rate, breathing rate, tidal volume, minute ventilation, and activity using textile RIP sensors [535]. For asthma patients, these devices enable continuous lung sound monitoring, capturing adventitious sounds like cough, wheeze, and crackles. In addition, wearable devices for monitoring babies’ breath have great significance [536].

The integration of wearable respiratory sensors with the IoT and ML algorithms enhances their capabilities, enabling real-time monitoring and analysis of sensor data for personalized, accurate, and efficient health monitoring through comprehensive breath analysis [537]. Wireless multifunctional wearable respiratory monitoring systems facilitate daily portable monitoring of various respiratory parameters. Despite the advancements, challenges remain, including ensuring data accuracy, addressing privacy concerns, and conducting large-scale clinical trials [538]. Future directions involve developing self-powered sensors and exploring smart fabrics to enhance wearability and comfort.

### 5.4. Intraocular Pressure Monitoring in Glaucoma Management

Glaucoma is a continuing neurodegenerative disease and major cause for irreversible blindness in the world. The primary risk factor for glaucoma is elevated intraocular pressure (IOP), which demands continuous monitoring for effective management. However, conventional methods, like Goldmann applanation tonometry, provide just single-point measurements and fail to record daily IOP fluctuations, which are critical for knowing the disease progression [539]. In this quest, the use of wearable and implantable BioMEMS-assisted IOP monitoring devices make it easier to diagnose and manage glaucoma by continuous and real-time tracking of IOP, cut down frequent clinical visits, and enhance treatment outcomes.

One of the major devices is smart contact lenses, which utilize microfluidic, piezoresistive, capacitive, or optical phenomena to detect changes in corneal curvature induced by IOP variations [540]. These sensors are minimally invasive and able to transmit real-time data wirelessly to mobile devices for remote monitoring [541]. For example, the Triggerfish^®^ contact lens is an FDA-approved device that can track continuously over 24 h, offering great information on nocturnal IOP patterns, which cannot be detected in routine clinical settings. Other futuristic designs include nanostructured materials as well as hybrid sensors for enhancing sensitivity and biocompatibility [542].

In comparison, implantable BioMEMS sensors facilitate direct measure of IOP with better accuracy. These devices are mostly capacitive [542], resonant [543], or optical in nature [544]. Moreover, those are implanted in many ocular locations, including the anterior chamber, sclera, or vitreous humor [545,546]. The transmission of power wirelessly using inductive coupling and relay of data through RF modules has further enhanced their efficiency and usability [547]. Some advanced implantable devices also utilize biomarker detection for a more comprehensive glaucoma assessment. Those advanced devices target inflammatory cytokines such as MMP-9 and IL-12p70 to improve the accuracy of diagnostics [548].

A positive encouragement in BioMEMS-based glaucoma care is accommodating automated drug delivery within wearable as well as implantable IOP sensors. Implementing such systems seems to have the potential to enhance the compliance of patients and optimize the outcomes of therapy [549]. Recent work related to nanoporous materials, microneedle patches, and hydrogel-based drug carriers has made intraocular drug release possible, thus reducing dependency on conventional eye drops [550]. Adoption of these devices in clinics is facing some challenges based on device size and long-term stability, as well as hurdles within the regulatory system [551]. However, advancements in technology such as flexible electronics, AI-driven data analysis, and wireless communication are expected to enhance their accuracy [552].

## 6. Regulatory, Global Standardization, and Societal Determinants

Despite the above-mentioned innovations in BioMEMS technologies, the path to widespread clinical adoption of these devices is influenced by several key determinants. These include complex regulatory processes, challenges with global standardization, and societal factors related to patient acceptance and usability. This section provides a glimpse at each of these determinants, highlighting the hurdles they present.

- **Complex Approval Processes:** The regulatory landscape for BioMEMSs is quite intricate and hence distinguished by lengthy approval processes. Regulatory bodies such as the U.S. Food and Drug Administration (FDA) and the European Medicines Agency (EMA) carry out thorough testing to ensure the functionality of such devices [553]. There are strict conditions for implantable BioMEMSs, or any other devices intended for applications such as heart monitoring or other telemetry, where balancing regulatory compliance and innovative ideas is fast becoming a significant challenge. Adequate time and resources must be invested in preclinical and clinical testing, which can lead to delays in product launching and increase costs [554]. Additionally, minimal standardized guidelines for new and upcoming BioMEMS technologies, such as organ-on-chip devices, makes the regulatory pathway more difficult [555].
- **Variability in Global Standards:** Global variability in regulatory standards is another major bottleneck for BioMEMS developers. Every region has a fixed set of requirements and extensive modifications, or additional testing is needed to fulfil those requirements. This variability in requirements can slow down the global scalability of BioMEMS technologies and increase developmental costs [556]. Coherence of regulatory frameworks, such as the International Medical Device Regulators Forum (IMDRF) initiatives, could make way for faster global acceptance. Joint efforts between regulatory agencies, industry, and academia can expedite better regulatory guidelines for BioMEMSs.
- **User-Friendly Design:** BioMEMS technologies can be successfully implemented in the long run if these methods are accepted by the patients. Many devices, particularly wearables and implantables, require high levels of comfort, ease of use, and inconspicuousness to ensure acceptance by patients. For example, heavy or intrusive devices may not be ideal for long-term use, and hence they become ineffective in chronic disease management [557]. In order to ensure this long-term usage, BioMEMS designers must focus on user-centered design principles. Flexible and lightweight materials, well-designed user-oriented form factors, and natural interfaces can significantly enhance patient comfort and acceptance. In addition, customizable devices specially made for individual needs could improve patient satisfaction and compliance [558].
- **Privacy and Data Security:** The amalgamation of BioMEMSs with digital health platforms can result in data privacy and security challenges [559]. Sophisticated devices dedicated to collection and transmission of sensitive health information are vulnerable to cyberattacks, data breaches, and unauthorized access. Secure data encryption coupled with secure communication protocols, and compliance with privacy regulations like HIPAA and the GDPR, will ensure privacy and data security and result in patient satisfaction [29].
- **Cultural and Psychological Barriers:** Cultural and psychological factors also play a key role in patient acceptance of BioMEMSs. For instance, people from different cultural backgrounds may have certain restrictions about implantable devices due to spiritual or moral beliefs. Likewise, patients with an apprehension of technology or operating procedures may be reluctant to consider BioMEMS-based solutions [560]. Overcoming these barriers necessitates proper planning in education and awareness campaigns to educate patients about the pros and cons of BioMEMSs. Healthcare providers must also be driven by initiative to address patient concerns and facilitate faith and confidence in these technologies [29].

## 7. Future Directions

As the BioMEMS field continues to progress, many promising opportunities are still in the early stages of exploration or development. This section focuses on several key future technological directions within BioMEMSs that have the potential to drive future innovations in the field.

- **Integration with Advanced Technologies:** The integration of BioMEMSs with advanced technologies like AI, ML, and the IoT holds immense promise [537]. AI and ML algorithms can analyze vast amounts of data generated by BioMEMS devices, enabling real-time decision-making and personalized medicine [561]. For instance, wearable BioMEMSs integrated with IoT can provide continuous patient monitoring and predictive analytics, facilitating early intervention for chronic diseases. Furthermore, the incorporation of edge computing into BioMEMS devices can reduce latency and improve energy efficiency. Edge-enabled BioMEMSs could process data locally, ensuring faster response times and enhanced privacy for sensitive health information. Research into secure and energy-efficient edge computing frameworks tailored for BioMEMS applications will likely be a focal point in the coming years [562].
- **Development of Multifunctional BioMEMSs:** Future BioMEMSs will likely transition from single-function devices to multifunctional systems capable of performing diverse tasks such as sensing, drug delivery, and therapeutic interventions [563]. For example, a BioMEMS device could monitor blood glucose levels and simultaneously deliver insulin, ensuring tighter glucose control for diabetic patients. Advances in microfabrication and materials science will be pivotal in enabling such multifunctionality. Additionally, there is a growing need for BioMEMSs that can integrate with complex biological environments [564]. Hybrid BioMEMSs combining electronic and biological components such as bio-hybrid actuators or bioelectronic interfaces could revolutionize the management of conditions like neurological disorders or organ dysfunction.
- **Advances in Materials for BioMEMSs:** Material innovation will play a critical role in shaping the next generation of BioMEMSs. Emerging materials like graphene, transition metal dichalcogenides (TMDs), and biocompatible hydrogels offer unique properties, such as high conductivity, flexibility, and biocompatibility. These materials can enhance device performance, longevity, and patient comfort [565]. Research into biodegradable and bioresorbable materials is particularly exciting. Devices made from such materials can safely degrade in the body after their intended function is complete, eliminating the need for surgical removal. This innovation is especially relevant for temporary implants or drug delivery systems [566].
- **Personalized and Precision Medicine:** The convergence of BioMEMSs and precision medicine presents a transformative opportunity to tailor healthcare interventions to individual patients. BioMEMS devices capable of analyzing genetic, proteomic, or metabolomic data could provide insights into a patient’s unique biological profile, enabling personalized treatment plans [567]. This approach is especially valuable in oncology, where BioMEMS-based platforms can identify biomarkers for specific cancer types and guide targeted therapies. Moreover, microfluidic BioMEMSs can facilitate organ-on-chip technologies, enabling researchers to study disease mechanisms and test potential treatments in a controlled environment [568]. These advancements could significantly accelerate drug discovery and reduce reliance on animal testing.
- **Enhancements in Point-of-Care Diagnostics:** Point-of-care (POC) diagnostics have already benefited greatly from BioMEMSs, but the future holds potential for even more compact, affordable, and accurate devices [569]. Future POC BioMEMSs could integrate nanoscale sensors and advanced signal processing to detect minute quantities of biomarkers, enabling early diagnosis of diseases such as cancer, infectious diseases, and neurodegenerative conditions [570]. Wearable and implantable BioMEMSs will also play a pivotal role in decentralized healthcare systems. These devices can provide continuous health monitoring, reducing the need for frequent hospital visits and improving patient outcomes. For instance, BioMEMS-enabled wearable sensors could monitor cardiovascular health in real time, alerting patients and physicians to potential risks.
- **Miniaturization and Power Efficiency:** The ongoing trend of miniaturization in BioMEMSs will continue, driven by advancements in nanotechnology and microfabrication techniques. Smaller devices offer several advantages, including reduced invasiveness, lower material costs, and enhanced portability [303]. However, miniaturization also presents challenges in terms of power consumption and device reliability. Future research will likely focus on energy-efficient power sources for BioMEMSs, such as energy harvesting from the human body. Technologies like piezoelectric nanogenerators and triboelectric energy harvesters could enable self-powered BioMEMSs, extending device longevity and reducing dependency on external power supplies [571].
- **Regenerative Medicine and Tissue Engineering:** BioMEMSs have significant potential in regenerative medicine and tissue engineering. Microfluidic devices can create 3D tissue constructs by precisely controlling cell deposition and nutrient delivery [572]. These constructs can be used for regenerative therapies, drug testing, or disease modelling. Moreover, BioMEMS-enabled bioprinters could revolutionize organ transplantation by producing patient-specific organs. Research into improving the resolution, speed, and scalability of BioMEMS-based bioprinting technologies will be critical in realizing this vision [573].

## 8. Conclusions

The integration of advanced materials, real-time biosensing, wireless connectivity, and therapeutic functionality has brought wearable and implantable BioMEMSs to the forefront of next-generation, data-driven healthcare. This review has provided a detailed look at recent progress in the field spanning the development of biocompatible, stretchable, and biodegradable materials to their practical applications in managing chronic diseases, cardiovascular conditions, respiratory disorders, and intraocular pressure. Wearable BioMEMS technologies, including flexible biosensors, bioelectronic patches, microneedle-based drug delivery systems, and motion sensors, are enabling non-invasive, continuous monitoring of physiological parameters and are reshaping healthcare into a more decentralized, personalized, and preventive model.

Implantable BioMEMSs have taken these capabilities a step further by offering long-term, in-body monitoring and intelligent, closed-loop therapeutic responses. These devices are being used in areas like neurostimulation for epilepsy and Parkinson’s disease, localized drug delivery for cancer, intracranial pressure regulation after traumatic brain injury, and temporary, bioresorbable implants for post-surgical care. When combined with AI and IoT technologies, BioMEMSs are paving the way for predictive diagnostics, real-time therapeutic adjustments, and remote patient care. For example, systems are being developed to detect heart attacks early by monitoring electrochemical signals, manage diabetes through automated insulin dosing, and accelerate healing with smart wound dressings that release drugs in response to infection or inflammation.

Despite these exciting developments, several challenges still need to be addressed. Regulatory systems often lag behind technological innovation, and the lack of globally unified standards can slow down product development and increase costs. In addition, human-centered issues like comfort, usability, long-term safety, and concerns around data privacy and ownership are critical in shaping whether these devices are accepted in real-world healthcare settings. Ethical questions, particularly around AI-driven decision-making and dissolvable or living implants, underscore the importance of building responsible frameworks as the field evolves.

Looking ahead, BioMEMSs are expected to become smarter, more multifunctional, and even fully biodegradable, and be capable of sensing and responding to complex biological changes while operating autonomously within connected digital health networks. Emerging applications include implantable bioelectronic systems for nerve modulation, organ-on-chip platforms customized for personalized drug testing, and next-generation neural interfaces for brain–machine communication in prosthetics or cognitive support. Smart ingestible capsules are also being designed to explore and analyze the gastrointestinal tract in real time, offering insights into gut health, metabolism, and even neurological conditions linked to the gut–brain axis.

The future may also see the merging of BioMEMSs with synthetic biology to create hybrid living devices that use engineered cells for sensing and therapeutic delivery. Meanwhile, integrating quantum sensors into BioMEMSs could dramatically improve the precision of molecular-level diagnostics, opening new frontiers in detecting diseases like Alzheimer’s or cancer in their earliest stages. Battery-free, energy-harvesting BioMEMSs powered by body motion or glucose are also on the horizon, promising longer device lifespans and less invasive procedures for patients.

Realizing this vision will require strong, ongoing collaboration among clinicians, engineers, materials scientists, regulators, and policymakers. Together, they can ensure these technologies are developed responsibly and made accessible to a wide range of healthcare settings, from advanced hospital systems to remote, underserved communities. As these innovations continue to evolve, BioMEMSs have the potential to fundamentally reshape modern medicine and move us towards a future where care is not only more precise and proactive but also more inclusive, patient-centered, and connected to the everyday lives of people around the world.

## Figures and Tables

**Table 1:** Comparison of the properties of different synthetic polymers [37,55,83,87,88,89,90,91,92,93,94,95,96,97,98,99,100,101,102,103,104,105,106,107,108].

Property	PDMS	Polyimide	Parylene C	PMMA	Polyurethane	Polypyrrole
Tensile strength (MPa)	3.51 to 5.13 MPa	78.3 ± 7.3	70	60–70	18	64
Elongation at break (%)	<100	7.4 ± 1.0	200	0.5–5	>140	5%
Young’s modulus (GPa)	360–870	2.5	4.5	3.3	3.6–88.8	>1
Mechanical flexibility	Very high	Moderate	Moderate	Low	Very high	Low
Hardness (Shore D)	41–43	1.21	Moderate	96	70.1	--
Density (g/cm^3^)	0.965	1.33	1.289	1.17	1.12	1.6
Water absorption (%) after 24 h	<0.05	--	0.1	--	--	--
Biodegradability	No	--	Yes	No	Yes	No
Biocompatibility	Excellent	Good (when pure)	Excellent	Good	Excellent (depends on formulation)	Moderate
Cytocompatibility	Yes	Yes	Yes	Yes	Yes	Yes
Glass transition temperature (°C)	−127	325	35–80	105	−35	65–95
Melting point (°C)	−49.9–−40	None, decompose instead melt	290	166	--	>300
Thermal conductivity (W/m·K)	0.2–0.27	8.04 × 10^−4^	1.0	0.22	0.19 ± 0.03	0.9
Dielectric constant (1 MHz)	2.3–2.8	3.4	2.95	3.5	4	--
Electrical conductivity (S/m)	4 × 10^−14^	Insulator	Insulator	1 × 10^−19^	Insulator	105

**Table 3:** Physical and mechanical properties of PCL [145,149,150].

Property	Values
Density (gm/cm^3^)	1.07–1.2
Average molecular weight (kDa)	50–130
Tensile elastic modulus (MPa)	250–440
Tensile strength (MPa)	10–30
Yield stress (MPa)	8–18
Melting temperature (°C)	56–65
Glass transition temperature (°C)	(−65)–(−60)
Strain at yield (%)	2–7
Strain at break (%)	80–800
Mechanical flexibility	High flexibility and toughness
Thermal conductivity (W/mK)	0.2–0.4
Electrical conductivity (S/m)	Non-conductive
Biocompatibility	Excellent

**Table 5:** Physical and mechanical properties of PHB and PHV [205,206,207].

Properties	PHB	PHV
Crystallinity (%)	60	53
Glass transition temperature (°C)	5	−5
Yield stress (MPa)	35	25
Elongation at break (%)	10	20
Mechanical flexibility	Brittle	More flexible than PHB
Elastic modulus (MPa)	1700	1200
Impact strength notched (KJ/mm^2^)	3	6
Impact strength without notch	Break	Break
Thermal conductivity (W/mK)	0.2–0.3	0.2–0.3
Electrical conductivity (S/m)	Non-conductive	Non-conductive
Biocompatibility	Excellent	Excellent

**Table 6:** Representative quantitative performance metrics of wearable BioMEMS biosensors.

Device Type	Application	Material/Platform	Sensitivity	LOD	Response Time	Reference
Wearable fiber-optic breath sensor	Respiration rate monitoring	Fiber-tip microcantilever + FP interferometer	0.8 nm/(m/s)	NA	300 ms (rise), 500 ms (fall)	[323]
Flexible printed biosensor (H_2_O_2_, AA)	H_2_O_2_ and ascorbic acid detection	Prussian Blue/reduced graphene oxide (PB/RGO) ink	31.65 μA·mM^−1^·cm^−2^ (H_2_O_2_)/58.7 μA·mM^−1^·cm^−2^ (AA)	1.78 μM (H_2_O_2_)/99 μM (AA)	~60 s	[371]
Dual-function wearable sweat biosensor	Glucose and uric acid monitoring	MWCNT-COOH/Prussian Blue/GOD ink-printed electrodes on gloves	1.64 µA/mM (UA), 1.32 µA/mM (glucose)	3.58 µM (UA), 9.10 µM (glucose)	60 s	[322]
Wearable sweat-based hormone biosensor	Cortisol, IL-6, IL-10, TNF-α, NPY (in sweat)	Nanoporous ZnO SPEs and graphene-based FETs (SLOCK, SWEATSENSER)	~30 kΩ–5 kΩ (impedance range)	IL-8 ~2 pg/mL (LOD)/Cortisol ~1 ng/mL	~1–5 min (depending on platform)	[319]
Hollow microneedle glucose patch	Continuous glucose monitoring (ISF)	Screen-printed GOx/PB/NiHCF sensor + PEEK microneedle + Nafion coating	−26.1 nA·mM^−1^ (ex vivo)	NA	2 min (ISF to readout)	[320]
Ultra-small wearable sweat biosensor	Glucose, lactate, Na^+^, K^+^ monitoring	Screen-printed flexible sensor array on PI + MS02 processor chip	2.05 nA/μM (glucose); 25 nA/mM (lactate); 43.76 and 57.38 mV/log [Na^+^]/[K^+^]	Not stated	~1–2 min	[321]
Self-powered TENG biosensor	Human motion and muscle monitoring;	Scotch tape–Al/PET triboelectric layer	Comparable to EMG sensors	NA	<100 ms	[372]
Printed core–shell biosensor	vitamin C, Trp, CK, and drug monitoring	MIP/NiHCF inkjet-printed nanoparticle ink	185 (CY), 253 (BU), 536 (MPA) in nA/mm^2^/decade	~5–10 µM (AA)	<1 min (DPV mode)	[373]
Wireless cortisol sweat sensor	Stress monitoring (sweat cortisol)	Laser-induced graphene + immunosensing (GS4)	~3.7 nA/mm^2^/ng/mL	0.08 ng/mL	<1 min (DPV)	[374]
Universal wearable sweat biosensor	Glucose, uric acid, lactate monitoring	Pt-NPs on N-doped mesoporous carbon/rGO (PNGO) + enzyme ink	15.33 µA·mM^−1^·cm^−2^ (glucose); 103.2 (UA); 219.1 (lactate)	10.83 µM (glucose); 3.21 µM (UA); 5.27 µM (lactate)	~1 min	[327]
Flexible SERS-based sweat sensor	Drug and biomolecule monitoring	Heart-shaped gold NP dimers on PDMS (F3S metasurface)	SERS EF: up to 10^11^	Single molecule	~1–2 min (Raman)	[343]
Wearable scalable SERS sensor	Sweat biomarkers, drugs, microplastics	Gold nanomesh on skin/fabric/plastic	SERS EF ≈ 10^8^	10 nM (R6G, AA)	~20 s (Raman scan)	[344]
Origami paper-based sweat biosensor	Glucose, lactate, UA, Mg^2+^, pH, cortisol	3D wax-patterned origami chip with enzymatic colorimetry + MIP sensor	R^2^ > 0.99 (colorimetry); electrochemical MIP for cortisol	1 nM (cortisol)	10–15 min (analyte-specific)	[345]
Soft epidermal microfluidic sensor	Sweat rate, pH, lactate, glucose, Cl^−^	Stretchable PDMS microchannels + colorimetric assay + NFC	0.2 mM (glucose), 0.3 mM (lactate), 0.1 mM (Cl^−^), 0.5 pH units	~1–2 mM (glucose); 1.5 mM (lactate)	<1 min (colorimetric)	[346]
Wireless self-powered acetone sensor	Breath acetone sensing for prediabetes	Chitosan–RGO film + triboelectric nanogenerator (WET)	27.89% @10 ppm acetone	NA	~1 min	[360]
Graphene–QD photodetector patch	HR, SpO^2^, RR, UV exposure monitoring	Graphene–PbS QD on PET/PI + NFC + Bluetooth	~10^5^ A/W responsivity	~3.7 × 10^−11^ W/cm^2^ NEI	50 µs (optical), <1 min (PPG)	[353]

**Table 7:** State-of-the-art devices for continuous health monitoring and diagnostics.

Device	Function	Technology	Clinical Application	Manufacturer
Abbott FreeStyle Libre	Glucose monitoring	Electrochemical biosensor	Diabetes management	Abbott Laboratories
Senseonics Eversense	Glucose monitoring	Fluorescence-based biosensor	Diabetes management	Senseonics
CardioMEMS HF System	Hemodynamic monitoring	MEMS-based wireless pressure sensor	Heart failure	Abbott
Synchron Stentrode	Brain–computer interface	Endovascular neural implant	Assistive communication for paralysis	Synchron
Withings Omnia	Comprehensive health monitoring	Integrated health tracking technologies	General health monitoring	Withings
Circular Smart Ring	Vital sign monitoring	Wearable sensors for HRV, sleep stages, blood oxygen levels	General health monitoring	Circular
Novosound’s Ultrasound Blood Pressure Monitor	Blood pressure monitoring	Ultrasound technology	Hypertension management	Novosound
Peri AI-Enabled Perimenopause Tracker	Perimenopause symptom tracking	AI-driven wearable	Women’s health	Peri
eDoctor Respiratory Health Monitor	Respiratory health monitoring	IoT-enabled sensors for vital signs	Respiratory health	TEKTELIC
eBeat Continuous Health Monitoring Device	Personalized vital sign monitoring	IoT-enabled sensors for real-time data transmission	Chronic disease management, elderly care	TEKTELIC

**Table 9:** A comprehensive summary of neurostimulation and bioelectronic implants.

Device	Function	Technology	Clinical Application	Manufacturer/Ref.
Medtronic DBS system	Deep brain stimulation	Electrical stimulation	Parkinson’s, essential tremor	Medtronic, Galway, Ireland
Vagus nerve stimulation (VNS) device	Neuromodulation	Electrical stimulation	Epilepsy, depression	LivaNova, London, UK
Spinal cord stimulator	Chronic pain relief	Electrical stimulation	Neuropathic pain	Boston Scientific, Marlborough, MA, USA
Magnetoelectric brain stimulator	Deep brain stimulation	Magnetoelectric metamaterial	movement disorders, psychiatric conditions	Robinson Lab (Company targeting 2025 FDA trial), Houston, TX, USA
Bioresorbable temporary pacemaker	Cardiac pacing	Bioresorbable electronics	Post-surgical cardiac care	Rogers Lab (Preclinical), Evanston, IL, USA
eCoin tibial neurostimulator	Overactive bladder management	Tibial nerve stimulation	Urological disorders	Valencia Technologies Corporation, Valencia, CA, USA
Boston Scientific Vercise DBS	Deep brain stimulation	Multi-target electrical stimulation	Dystonia, Parkinson’s	CE Mark (Boston Scientific), Marlborough, MA, USA
NEVRO HF10 therapy	Chronic pain management	10 kHz high-frequency stimulation	Chronic trunk/limb pain	NEVRO (FDA-Approved), Redwood City, CA , USA
Responsive neurostimulation (RNS) system	Seizure control	Closed-loop brain sensing + stimulation	Epilepsy	UCSF Epilepsy Center, San Francisco, CA 94143, USA
Optogenetic implant	Neural modulation	Light-activated ion channels	Neurological disorders	Research stage [488]
Ultrasound neuromodulation device	Non-invasive stimulation	Focused ultrasound waves	Chronic pain, depression	Research stage [489]
PrimeAdvanced SCS	Spinal cord stimulation	Rechargeable multi-waveform	Chronic pain	Medtronic, Galway, Ireland
Intellis™ neurostimulator	Adaptive pain relief	Closed-loop sensing + AI algorithms	Failed back surgery syndrome	Medtronic, Galway, Ireland
Biohybrid regenerative implant	Tissue repair + electrical stimulation	Cell-embedded conductive scaffolds	Cardiac/neural regeneration	Research stage [490]

## Data Availability

The authors will provide available data or assist in finding them in the databases.
